# Unraveling the Role of Lignin Oligomers Functionality for the Synthesis of Nonisocyanate Polyurethanes

**DOI:** 10.1002/cssc.70953

**Published:** 2026-08-07

**Authors:** Nathan Wybo, Hugo Lilti, Dorothée Laurenti, Christophe Geantet, Antoine Duval, Luc Avérous

**Affiliations:** ^1^ BioTeam/ICPEES‐ECPM UMR CNRS 7515 Université de Strasbourg Strasbourg France; ^2^ IRCELYON UMR CNRS 5256 Université Lyon 1 UCBL Villeurbanne France; ^3^ Soprema Strasbourg France

**Keywords:** bio‐based, depolymerization, hydrothermal depolymerization, lignin, nonisocyanate polyurethanes, polyhydroxyurethanes, reductive catalytic depolymerization

## Abstract

Lignins are the main source of aromatic compounds in nature. Their depolymerization most often leads to oligomeric products, which lack proper valorization. In this study, two lignins (Kraft and organosolv) were subjected to two depolymerization methodologies, leading to a total of six lignin samples, which were characterized and functionalized to cyclocarbonate (CC) building blocks through a green and optimized one‐pot procedure. The lignin oligomers obtained by depolymerization were compared to the raw lignins for the synthesis of safe and sustainable polyhydroxyurethane (PHU) thermosets by aminolysis with lignin contents between 28 and 34 wt%. The synthesized PHU materials displayed a wide range of mechanical properties and behaviors, with a tensile modulus ranging from 50 to 1400 kPa, and elongation at break ranging from 46% to 217%. While lignin depolymerization had a negligible effect on the polymerization kinetics, it led to softer and more flexible materials at equivalent lignin contents. Interestingly, it was found that the functionality of the lignin‐CC building blocks is the main factor affecting the crosslinking density, and consequently the mechanical properties of the materials. Other factors such as the lignin type (e.g., Kraft vs. organosolv) and the depolymerization method (e.g., hydrothermal vs. reductive) did not have a strong influence on the final material properties. These different points establish a strong foundation for the understanding of “structure–properties” relationships in lignin‐based materials, a key point toward the valorization of lignin into renewable, safe, and high‐performance materials.

## Graphical abstract

This study reports the synthesis of bio‐based nonisocyanate polyhydroxyurethanes from crude lignins and depolymerized lignin oligomers. The structure–properties relationships were largely explored and highlighted the key role of lignin functionality on the physical properties of the materials.

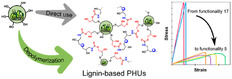

## 1 Introduction

Lignin makes up approximately 25% of woody biomass and represents the largest renewable source of aromatic compounds. It is a polyphenolic biopolymer, obtained as a byproduct of the pulp and paper and bioethanol industries [1, 3]. However, more than 90% of its worldwide production, estimated at around 100 Mt/y, is burnt in boilers for energy recovery [4, 5]. To enhance the economic and environmental sustainability of lignocellulosic biorefineries, lignin needs to find integrated valorization pathways.

Lignin is a promising feedstock for producing bio‐based aromatic compounds, which usually have high value and extended applications (pharmaceuticals, fuels, polymers, etc.). However, depolymerizing lignin to aromatic monomers is a challenging task [6, 8]. The yield is directly correlated with the quantity of cleavable bonds in the lignin structure, mostly β‐O‐4 ether linkages [9, 11]. With technical lignins, such as Kraft, soda, or some organosolv lignins, whose contents in β‐O‐4 are rather low [12], depolymerization mostly leads to the recovery of an oligomeric fraction (oLig) [11]. An appealing application for these complex mixtures of polyphenolic compounds is the synthesis of bio‐based aromatic polymers [11, 13, 16].

oLigs offer different advantages for the synthesis of valuable polymers. Their aromaticity can bring rigidity, heat resistance, hydrophobicity, and barrier properties [17, 20], while their high functionality allows for the production of thermoset polymers with improved mechanical and heat resistance. Compared to crude lignins, oLigs usually exhibit lower molar mass and dispersities, which can improve solubility and dispersion in the polymerization media, while lowering viscosity at high bio‐based contents [21, 23]. Depending on the depolymerization methodology, building blocks with increased hydroxyl contents and reactivity can also be produced, which can lead to increased crosslinking and rigidity.

Recent studies on epoxy systems show that oLigs can substitute for fossil‐based bisphenols without compromising the mechanical properties [15, 16, 24]. Unlike conventional bisphenols, such as bisphenol A, oLigs contain methoxy groups, which enhance interchain hydrogen bonding and lower aromatic π–π stacking [25, 26], leading to materials with higher glassy modulus but lower *T*_g_ [27, 28].

oLigs have also been studied in polyurethane (PU) systems [11, 13]. However, PUs are synthesized with isocyanates, which are toxic and sensitizing compounds [29, 30], responsible for most of the environmental impact of PUs [31]. The development of nonisocyanate PUs (NIPUs) thus emerged as a safer and more sustainable alternative to conventional PUs [32, 34]. They offer large opportunities in applications such as foams, adhesives, coatings, elastomers, and biomaterials [20, 35, 39]. The main synthetic route to generate NIPUs is the aminolysis of cyclocarbonates (CCs), which generates polyhydroxyurethanes (PHUs) [33, 34, 40]. The use of oLigs in bio‐based NIPUs has been relatively unexplored. A prior work by Quinsaat et al. introduced oLigs functionalized to CC into epoxy systems to form hybrid epoxy‐PHU materials, showing that small amounts of oLig‐based hydroxyurethanes could improve the properties of the materials [41]. More recently, PHU materials were synthesized from oLigs obtained by reductive catalytic fractionation (RCF) of biomass [42, 43]. However, these studies face several limitations, including the use of low amounts of lignin, extensive modifications of the oligomers, and the use of large quantities of solvents. Additionally, they do not evaluate the influence of the structure of the lignin and its oligomers from different depolymerization processes on the material properties. Therefore, the underlying phenomena affecting the PHU properties are still ignored.

This study aims at bridging the knowledge gap on the interest of using oLigs in PHUs, rather than neat lignins. For this purpose, two types of lignins (Ligs) were depolymerized through hydrogenolysis and hydrothermal depolymerizations, yielding four distinct oLig fractions. Ligs and oLigs were subsequently functionalized into CC building blocks through an optimized and green one‐pot process using safe carbonates. The resulting Lig‐CCs and oLig‐CCs were polymerized with a diamine based on poly(ethylene glycol) to yield PHUs, which were characterized by Fourier transform infrared spectroscopy (FTIR), thermogravimetric analysis (TGA), differential scanning calorimetry (DSC), swelling tests, dynamic mechanical analysis (DMA), and tensile tests to highlight the impact of lignin depolymerization on the material properties.

## 2 Experimental Section

### 2.1 Materials

Beechwood organosolv Lignin (HW‐OL) was provided by TNO (Netherlands). It was obtained at pilot scale through the aqueous acetone Fabiola process. Details on the extraction process and lignin characterization can be found elsewhere [44, 45]. Softwood Kraft lignin (SW‐KL) was purchased as Lineo from Stora Enso (Finland). It was extracted from black liquor through acidic precipitation in the LignoBoost process.

Glycerol carbonate (Jeffsol GC) was kindly provided by Huntsman Corporation (Germany). Poly(ethylene glycol) diglycidyl ether of *M*_n_ = 526 g/mol (PEG‐dGE), 1,5,7‐triazabicyclo [4.4.0]dec‐5‐ene (TBD), hydrochloric acid (37% in water), chromium(III) acetyl acetonate (Cr(acac)3), cholesterol, 1,3,5‐trioxane, and 2‐chloro‐4,4,5,5‐tetramethyl‐1,3,2‐dioxaphospholane (Cl‐TMDP, 95%) were bought from Sigma‐Aldrich. Sodium iodide (NaI) was bought from Acros Organics. Dimethyl carbonate (DMC), sodium chloride (NaCl), potassium carbonate (K_2_CO_3_), 3,4,5‐trichloropyridine (TCP), 35% ammonia solution, and anhydrous magnesium sulfate (MgSO_4_) were purchased from Fisher Scientific. Deuterated dimethylsulfoxide (DMSO‐*d*_6_) and deuterated chloroform (CDCl_3_) were purchased from Eurisotop. Acetone and dichloromethane were purchased from Carlo Erba Reagents. Pyridine was obtained from Alfa Aesar.

### 2.2 Lignin Depolymerization by Hydrothermal

40 g of lignin was loaded into a 300 mL Parr batch reactor with 200 mL of water and 8 g of sodium chloride ([NaCl]: 40 g/L). The reactor was closed and flushed with nitrogen for 2 min, then filled with 10 bar N_2_ under stirring and emptied (3 times) to remove remaining air. The reactor was heated under 1 bar N_2_ to 320 °C with a heating rate of 10 °C/min and a stirring rate of 700 rpm. After 1 h at the targeted temperature, the reactor was cooled. The liquid aqueous fraction was separated from the solid, which was washed with tetrahydrofuran (THF) to recover the soluble oligomeric fraction. THF was then removed by rotary evaporation, and the obtained solid was dried under vacuum at 50 °C overnight (45 wt% yield based on initial lignin).

Samples depolymerized using this method are named HTL‐SW‐KL and HTL‐HW‐OL, depending on the initial lignin used.

### 2.3 Lignin Depolymerization by Hydrogenolysis

40 g of lignin was loaded into a 300 mL Parr batch reactor with 200 mL of isobutanol as the solvent and 4 g of sulfided CoMoS/Al_2_O_3_ catalyst (CoMoS). The CoMoS catalyst was obtained by sulfidation of the commercial oxide precursor (provided by Axens, shape as an extrudate length of 2–10 mm; diameter of 1.2 mm), as described elsewhere [46]. After removing air by flushing with N_2_, as in the hydrothermal depolymerization, the temperature was set to 280 °C with a heating rate of 10 °C/min. When the temperature was reached, the pressure was set to 90 bar by adding hydrogen (around 50 bar of H_2_). After 2 h of reaction, the reactor cooled down. The isobutanol‐soluble products were recovered by filtration, and the solid fraction was washed with THF. The THF‐soluble and isobutanol‐soluble products were combined, and both solvents were removed together by rotary evaporation. The recovered products were finally dried at 50 °C in a vacuum oven overnight (88 wt% yield based on initial lignin).

Samples depolymerized using this method are named H_2_‐SW‐KL and H_2_‐HW‐OL, depending on the initial lignin used.

### 2.4 Lignin‐Based CCs (Lig‐CC and oLig‐CC) Synthesis

Lignins were modified in a one‐pot process. 20 g of lignin was mixed with GC (10 eq. with respect to the total hydroxyl content of the lignin), and K_2_CO_3_ (0.1 eq. with respect to the total hydroxyl content of the lignin). After full dissolution of the lignin, the mixture was reacted at 130 °C for 6 h. The reaction mixture was then cooled to 90 °C, and DMC (5 eq. with respect to the total hydroxyl content of the lignin) was added slowly. The reaction was continued at 90 °C under reflux for 4 h. Lignin was then precipitated by diluting the reaction mixture 8 times in a 0.01 M HCl solution. The solid was filtered, washed 4 times with water, and dried under vacuum at 40 °C overnight to yield Lig‐CC. Mass yields ranged from 137% to 149% (Table S5).

A similar procedure was performed on oLig to generate oLig‐CC. The reaction with GC was performed for 18 h, and the second step with DMC for 6 h. Mass yields ranged from 82% to 154% (Table S5).

### 2.5 PEG‐Based Bis‐CC (PEG‐bCC) Synthesis

PEG‐bCC was prepared according to a previously described procedure [47]. 80 g of PEG‐dGE was mixed with 3.8 g of NaI (0.07 eq.) in a pressurized reactor. The reactor was flushed with argon and pressurized to 3 bar with CO_2_. The mixture was reacted under agitation at 90 °C for 10 h. Then, the reaction mixture was extracted with 300 mL of dichloromethane and washed three times with brine. The organic phase was recovered and dried over anhydrous MgSO_4_ and evaporated under vacuum to yield PEG‐bCC (93% yield).

### 2.6 PEG‐Based Bis‐β‐Hydroxyamine (PEG‐bNH_2_) Synthesis

50 g of PEG‐dGE was dissolved in 288 mL of 25% NH_3_ solution (13.2 mol/L), corresponding to 20 eq. of NH_3_ per epoxy. The mixture was placed in a sealed reactor flushed with argon, and agitated at 100 °C for 6 h. The ammonia solution was then evaporated under reduced pressure to yield PEG‐bNH_2_ (100% yield).

### 2.7 Prepolymer (Prepo‐bNH_2_) Synthesis

47.5 g of PEG‐bNH_2_, 19.0 g of PEG‐bCC (CC/NH_2_ ratio of 0.4), and 1.09 g of TBD (5 mol% with respect to NH_2_ groups) were reacted at 80 °C under argon with mechanical agitation. The reaction was stopped when the CC band in the FTIR (1790 cm^−1^) had fully disappeared (approximately 1.5 h). Prepo‐bNH_2_ was used as is.

### 2.8 Nonisocyanate PHUs Synthesis

Lig‐CC or oLig‐CC, Prepo‐bNH_2_ (NH_2_/CC ratio of 1.0), and acetone were mixed thoroughly with a mortar at room temperature until a homogeneous mixture was obtained. The reaction mixture was then cured under a gentle argon flux at 80 °C for 4 h, then at 120 °C for 4 h.

With Lig‐CC, the material was then hot pressed into a sheet (100 × 50 × 1 mm) for 2 h at 160 °C under 150 bar (7.5 tons). With oLig‐CC, to prevent the material from flowing, the reaction mixture was cured at 160 °C for 2 h before being pressed for 30 min at 140 °C under 150 bar.

All the materials were then postcured at 160 °C for 4 h.

### 2.9 Characterizations

FTIR spectra were obtained with a Nicolet 380 spectrometer (Thermo Electron Corporation) equipped with an attenuated total reflectance (ATR) diamond module. Each analysis was acquired with 32 scans in the 4000–400 cm^−1^ range at room temperature.

^1^H, ^19^F, and ^31^P NMR spectra were acquired with a BRUKER Advance III HD—400 MHz instrument with a BBFO probe. ^13^C NMR spectra were acquired with a BRUKER Advance III HD—500 MHz spectrometer with a CryoProbe BB(F)O Prodigy.

^1^H NMR were performed in DMSO‐*d*_6_, with 16 scans using a zg30 sequence with a 1 s relaxation delay. For quantitative ^1^H NMR, approximately 25 mg of sample was dissolved in 0.6 mL of DMSO‐*d*_6_. Then, 100 μL of 0.5 M TCP in DMSO‐*d*_6_ was added. 16 scans were recorded using a zg30 sequence with a 30 s relaxation delay.

Quantitative ^13^C NMR were performed following a similar protocol as in the literature [48]. Approximately 80 mg of sample was dissolved in 0.6 mL of DMSO‐*d*_6_. Then, 100 μL of a 0.5 M TCP and 25 mg/mL Cr(acac)_3_ solution in DMSO‐*d*_6_ was added. 4096 scans were recorded using a zgig30 inverted gated proton‐decoupled sequence with a 4 s relaxation delay.

Quantitative ^31^P NMR of lignins was performed following the standard literature protocol [49]. About 30 mg of sample was dissolved in 450 µL of pyridine/CDCl_3_ 1.6/1 mixture. Then 100 µL of a Cr(acac)_3_ 5 mg/mL and cholesterol (internal standard) 0.1 M solution and 75 µL of Cl‐TMDP were added. The vessel was closed and agitated for 2 h before analysis. 128 scans were recorded using a zgig30 sequence with a 15 s relaxation delay.

Quantitative ^19^F NMR of PEG‐bNH_2_ and Prepo‐bNH_2_ was performed following the protocol previously reported by our team [50]. About 30 mg of sample was mixed with 0.6 mL of DMSO‐*d_6_*, 40 µL of *para*‐(trifluoromethyl)benzaldehyde and 90 µL of a triethylamine/2,2,2‐trifluoroethanol (8/1, v/v) mixture and around 15 beads of molecular sieves. The vessel was closed and agitated for 24 h at room temperature before analysis. 16 scans were recorded with a 30 s relaxation delay.

Size‐exclusion chromatography (SEC) was performed using an Acquity APC apparatus from Waters, with THF as the eluent (0.6 mL/min) at 40 °C. Detection was performed by an Acquity ultraviolet transmission detector (TUV). Three APC XT columns (4.6 × 150 mm) of different porosity (450, 200, and 45 Å) were connected. All samples were acetylated before analysis as described in the literature [50], to maximize their solubility in THF and limit their interactions with the columns. The calibration was based on polystyrene (PS) standards. The detector response was normalized based on the total integration of each signal.

TGA was performed on a Mettler Toledo TGA 2 STARe system. Samples of approximately 3 mg were heated in an alumina crucible from room temperature to 700 °C at a rate of 20 °C/min under a N_2_ atmosphere (25 mL/min). *T*_5%_ was determined as the temperature at which 5% weight loss occurred. TGA derivatives (DTG) were used to determine the temperatures at the maximum rate of mass loss (*T*_d_).

DSC measurements were recorded on a TA Instruments Discovery DSC‐25 apparatus under a 50 mL/min dry nitrogen flow. 3 mg samples were placed in aluminum pans, with an empty pan as reference. To erase the thermal history, the samples were first heated to 105 °C for 10 min. They were then cooled down to −60 °C. Finally, the samples were heated up to 200 °C at a 10 °C/min rate. Glass transition temperatures (*T*_g_) were measured at the inflection point of heat flow during the last heating ramp.

DMA was performed using a Discovery HR‐3 Hybrid Rheometer by TA Instruments. A 2 mm ×1 mm sheet of sample was placed vertically in a rectangular torsion geometry. The sample was heated at 3 °C/min from −50 to 150 °C with a 0.01% strain at a frequency of 1 Hz. *T*_α_ was recorded at the maximum of the tan δ curves.

Uniaxial tensile tests were performed on a Discovery HR‐3 Hybrid Rheometer by TA Instruments with a tension apparatus (50 N load cell), in a room set at 27 °C with a constant crosshead speed of 20 mm/min. Experiments were performed on a set of three samples with dimensions of approximately 30 × 4.6 × 1 mm^3^ until failure. Young's modulus (*E*), tensile strength (*σ*), and elongation at break (*ε*) were recorded. The reported tensile curves were selected to be representative of the average values.

The swelling ratio (SR) of the materials was measured on triplicate samples (approximately 200 mg) after immersion in water or acetone for 72 h. Equation (1) was used to obtain the SR, with *m*_swelled_ the mass of the sample after immersion, and *m*_final_ the mass of the sample after drying at 40 °C under vacuum overnight. Gel fractions (GF) were calculated using Equation (2).

(1) $$\mathrm{SR}\;=\;\frac{m_{\text{swelled}}-m_{\text{final}}}{m_{\text{final}}}\;\times 100$$

(2) $$\mathrm{GF}\;=\;\frac{m_{\text{final}}}{m_{\text{initial}}}\;\times 100$$

## 3 Results and Discussion

### 3.1 Characterization of the Depolymerized Lignins

**FIGURE 1:**
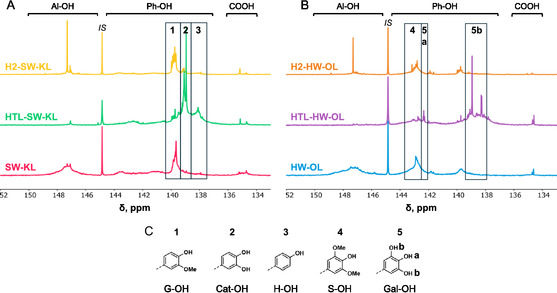
^31^P NMR of Ligs and oLigs. (A) SW‐KL and its derivatives. (B) HW‐OL and its derivatives. (C) Assignation of the chemical groups.

Two distinct lignins were depolymerized to obtain a set of oLigs with different chemical structures and later evaluated their impact on the properties of PHUs. A SW‐KL rich in guaiacyl (G) units and a hardwood organosolv lignin (HW‐OL) containing mostly syringyl (S) units were treated by reductive catalytic (H_2_‐Depol) and hydrothermal (HTL‐Depol) depolymerizations. In total, six different samples (two initial Ligs and four oLigs) were analyzed by FTIR, ^1^H NMR, quantitative ^31^P NMR, ^1^H–^13^C HSQC NMR, SEC, DSC, and TGA to assess their chemical structure, molar mass distribution, and thermal properties (Figures 1 and S1–S10, Tables S1 and S2).

HTL‐Depol involved heat treatment of lignin in a 4 wt% NaCl aqueous solution at 320 °C. SEC of HTL‐SW‐KL and HTL‐HW‐OL revealed apparent *M*_n_ of 740 and 860 g/mol, respectively, with dispersities of 2.0 and 1.9, showing a successful depolymerization to oligomers of a few aromatic units (Table 1, Figure S3 and Table S2). A strong increase in the phenolic content, up to 8.1 and 7.6 mmol/g for HTL‐SW‐KL and HTL‐HW‐OL, respectively, was measured by ^31^P NMR. For HTL‐SW‐KL, new peaks are observed at 138.9–139.0 and 138.1 ppm (**2** and **3** in Figure 1). They can be assigned to catechols and *p*‐hydroxyphenyl, respectively [49, 51, 52], indicating that demethylation of methoxy groups and demethoxylation occurred in G units. For HTL‐HW‐OL, peaks at 142.3, 138.9, and 138.3 ppm, corresponding to galloyl structures, are observed (**5** in Figure 1) [51, 52], originating from demethylation in S units. Catechols coming from demethylation of G units are also probably present, although they overlap with the galloyl groups. Aliphatic alcohols are largely lost during the process, making HTL‐Depol oLigs almost purely phenolic compounds (Table 1).

**TABLE 1:** Analysis of Ligs and oLigs by ^31^P NMR, SEC, TGA, and DSC.

Lig/oLig	^31^P NMR		SEC		TGA			DSC
	PhOH, mmol/g	AlOH, mmol/g	*M*_n_, g/mol	*Ð*	*T*_5%_, °C	*T*_d_, °C	Residue at 700 °C, %wt	*T*_g_, °C
SW‐KL	3.94	2.03	1347	3.3	228	398	39	120
HW‐OL	2.77	1.80	1582	2.7	241	375	38	141
HTL‐SW‐KL	8.07	0.09	737	2.0	182	—	67	39
HTL‐HW‐OL	7.56	0.12	861	1.9	174	—	66	59
H_2_‐SW‐KL	5.01	1.39	507	1.7	173	400	20	12
H_2_‐HW‐OL	4.11	0.98	561	1.5	163	387	19	26

*Note:* *T*_5%_, temperature at 5 wt% loss from TGA. *T*_d_, temperature of the maximum degradation rate from TGA. *T*_g_, glass transition temperature from DSC. Abbreviations: AlOH, alcohols; PhOH, phenols.

H_2_‐Depol was performed in isobutanol using a CoMoS catalyst at 280 °C under 90 bar of hydrogen. More details about the procedure and catalyst choice can be found in a recent publication [46]. This methodology led to lower apparent *M*_n_ (507 and 561 g/mol) and *Ð* (1.7 and 1.5). H_2_‐Depol also increased the phenolic contents to 5.01 and 4.11 mmol/g, but, unlike for HTL‐Depol, demethylation of the methoxy groups was limited. Aliphatic alcohols were also largely lost during H_2_‐Depol, but the samples contained traces of isobutanol used as the solvent (147.3 ppm).

^1^H–^13^C HSQC of the Ligs and oLigs confirms the evolution in phenolic structures during depolymerization (Figures S4– S7). In particular, oLigs obtained by H_2_‐Depol show aromatic signals close to those of the original Ligs, while HTL‐Depol oLigs have a heavily modified structure. With both methodologies, interunit linkages (mainly β‐O‐4, β‐β, β‐5) disappear, and chain ends (methyl, ethyl, propyl, and *n*‐propanol) appear more intense in HSQC.

The thermal properties of oLigs were analyzed by TGA and DSC (Table 1, Figures S8–S10). oLigs had significantly lower glass transition temperature *T*_g_ (between 12 and 59 °C) than Ligs (120 and 141 °C), because of their higher mobility due to their lower molar mass. They presented lower temperatures at 5% weight loss (*T*_5%_), probably due to the formation of low molar mass volatile compounds. oLigs obtained by H_2_‐Depol showed a similar mass loss to the Ligs, with a main degradation temperature *T*_d_ occurring at around 400 °C, as expected from their similar structures. However, HTL‐Depol oLigs had a significantly different degradation behavior, with a steady mass loss and a high quantity of residues (66%) at 700 °C.

Overall, both depolymerization methodologies led to oligomer mixtures (oLig) with significantly reduced molar masses and dispersities. HTL‐Depol generated a strong increase in phenolic groups caused by the demethylation of G and S units, leading to catechol and gallol structures. On the other hand, H_2_‐Depol preserved the methoxy groups of the lignins and led to a more moderate increase in phenolic groups thanks to the cleavage of ether interunit linkages.

### 3.2 Lignins Functionalization to Polycyclocarbonates

**SCHEME 1:**
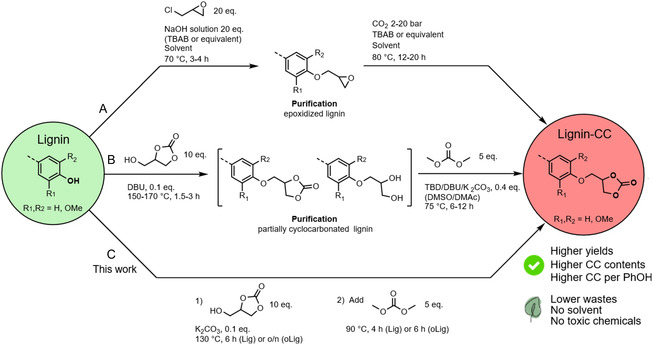
Reaction schemes for the functionalization of Ligs and oLigs to CC building blocks. (A) ECH pathway [53, 55, 61]. (B) GC‐DMC pathway [48, 56, 58]. (C) One‐pot GC‐DMC pathway performed in this work.

To synthesize PHUs, Ligs and oLigs were first functionalized to CC building blocks. As seen in Scheme 1, two main functionalization pathways have been reported in the literature: (a) lignin epoxidation with epichlorhydrin (ECH) followed by carbonate formation with CO_2_ [43, 53, 55], and (b) oxyalkylation to a diol with GC, followed by ring closing with dimethylcarbonate (DMC) [48, 56, 58]. The latter can be advocated as safer and more sustainable, as it increases mass yields (63% to 106%), significantly lowers the E factor associated with organic compounds (130–13), and avoids the use of toxic and potentially carcinogenic ECH (Table S3) [57]. GC and DMC can also be derived from CO_2_ following green procedures [59, 60]. The GC + DMC methodology was previously reported as two‐steps procedure, with purification of the intermediate product. Herein, we disclose an improved one‐pot methodology, avoiding the use of solvents and toxic chemicals. Ligs and oLigs were first reacted with GC under K_2_CO_3_ catalysis. DMC was then added to the reaction mixture to convert the formed diols into CC, yielding Lig‐CCs and oLig‐CCs (Scheme 1C). Overall, this procedure increases mass yields, lowers *E* factors below 5, and significantly limits water consumption for purification (Table S3).

**FIGURE 2:**
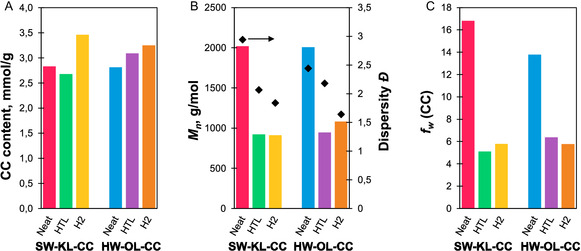
Characterizations of the functionalized lignins. (A) CC content as measured by quantitative ^13^C NMR. (B) Apparent molar mass *M*_n_ and dispersities *Ð* of acetylated Lig‐CCs and oLig‐CCs as measured by SEC. (C) Weight‐average functionality in CC *f*_w_ (CC) estimated from Equation (3).

**SCHEME 2:**
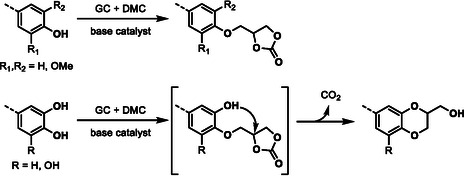
Reactions of phenols leading to CC and catechols/gallols leading to benzodioxanes [62].

Quantitative ^31^P NMR showed that 100% of phenols (PhOH) were converted under the reaction conditions (Figure S12 and Table S4). FTIR confirmed the formation of CC groups, with the appearance of a new characteristic band at 1790 cm^−1^ (Figure S13). The CC contents were evaluated by quantitative ^13^C NMR by integration of the characteristic peak between 154.5 and 155.5 ppm (Figure S14). For Ligs and H_2_‐oLigs, the CC contents range from 2.8 to 3.5 mmol/g, corresponding to the conversion of 70% to 100% of the initial PhOH groups (Figure 2A and Table S4). In the case of HTL‐oLigs, the CC contents are in the same range (2.7 and 3.1 mmol/g), which corresponds to only 33% and 40% of the initial PhOH content. ^13^C NMR reveals that catechol and galloyl structures, which are omnipresent in HTL‐oLigs, were converted to benzodioxanes with GC, as previously reported (Scheme 2) [62].

Considering the mass increase during the reaction (grafting of CC groups), CC contents lie between 1.0 and 1.4 times the expected values for total PhOH conversion with Ligs and H_2_‐oLigs (Table S5). This shows that the conversion of PhOH to CC is quantitative, and that minor reactions can also graft CC onto aliphatic alcohols [63]. Our optimized methodology significantly increases the CC content of the products compared to previous literature, which reached only up to 2.05 mmol/g CC (Table S6) [48, 53, 56, 57]. This can be explained by the more selective reaction of GC compared to ECH with lignin, and the lower temperatures used.

As a consequence of the functionalization, the molar masses of Lig‐CCs and oLig‐CCs slightly increased compared to Ligs and oLigs (Figures 2B, S15, and Table S7). SEC confirms that crosslinking did not occur, as the increase in *M*_n_ is limited, and *Ð* is unaffected by the transformation. This result is particularly interesting, as previous reports using GC + DMC methodology evidenced crosslinking by linear carbonates [42, 57]. Here, signals corresponding to these structures (155.8 and 156.2 ppm) were not detected by ^13^C NMR (Figure S14). The absence of such crosslinking could arise from the lower temperatures used in our one‐pot methodology (130 °C compared to 150–170 °C).

An important parameter to understand the behavior of Lig‐CCs and oLig‐CCs in PHU synthesis is the average functionality, i.e., the average number of reactive groups per molecule. To describe gelation in crosslinked systems, the weight‐average functionality *f*_w_ has to be considered [64, 65]. It can be estimated from the CC content measured by ^13^C NMR ([*CC*] in mol/g) and the apparent *M*_w_ measured by SEC (in g/mol), according to Equation (3).

(3) $${\bar{f}}_{w}(\mathrm{CC})=[\mathrm{CC}]\times {\bar{M}}_{w}$$

However, this estimation is only indicative, since *M*_w_ was obtained by SEC with standard calibration, which can lead to significant deviations from absolute molar masses [66, 67]. In general, SEC with standard calibration tends to underestimate the absolute *M*_w_ [68, 69], meaning that the calculated *f*_w_ are also probably underestimated. From the apparent *M*_w_ and CC contents, Lig‐CCs were expected to have 14–17 CC per molecule, while oLig‐CCs would have CC functionalities ranging from 5 to 6 per molecule (Figure 2C).

Thermal analysis by TGA shows that all modified lignins, including HTL‐oLig‐CC, have similar thermal degradation behavior, with a *T*_5%_ ranging from 227 to 277 °C and main degradation between 392 and 415 °C (Figures S16, S17 and Table S7). This confirms that ether bond cleavage is the main phenomenon leading to thermal weight loss in lignins. The functionalization led to a decrease in *T*_g_ of Lig‐CCs and oLig‐CCs as compared to the parent lignins, as a consequence of the loss of hydroxyl groups (Figure S18 and Table S7).

To summarize, Ligs and oLigs were successfully modified to CC building blocks through an optimized one‐pot procedure. All phenols were readily grafted with CC, leading to CC contents up to 3.5 mmol/g, well above previous published works. The functionalization did not generate crosslinking, thus preserving the molar mass distribution of Ligs and oLigs. Consequently, the lignin‐based CC show a wide range of average functionality, between 5 and 17 CC groups per molecule.

### 3.3 PHU Synthesis

**SCHEME 3:**
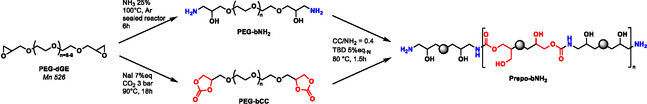
Reaction scheme of the synthesis of Prepo‐bNH_2_.

An amine‐terminated PHU prepolymer with a PEG backbone (Prepo‐bNH_2_) was first prepared. A PEG‐based structure was chosen because of the high compatibility between lignin and PEG [70, 71], which ensures the obtaining of homogeneous materials. Prepo‐bNH_2_ was synthesized by reaction of PEG‐bNH_2_ with PEG‐bCC at a CC/NH_2_ molar ratio of 0.4 (Scheme 3). This ratio was tailored to obtain PHUs with a lignin content around 30 %wt by reacting the prepolymer with Lig‐CCs or oLig‐CCs at an NH_2_/CC ratio of 1.0, allowing good processability.

Both PEG‐bNH_2_ and PEG‐bCC were synthesized quantitatively from PEG‐dGE. The prepolymer synthesis was performed at 80 °C with TBD as catalyst and led to quantitative conversion of CC in 1.5 h as evidenced by FTIR (Figure S13). Quantitative ^31^P NMR allowed the quantification of hydroxyl groups originating from PEG‐bNH_2_ and the newly formed hydroxyurethanes, which confirmed the structure of the product and its amine content of 1.37 mmol/g (Figure S22).

**SCHEME 4:**
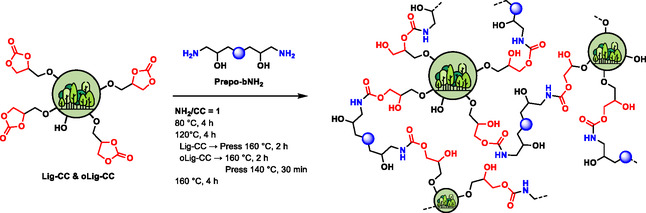
Reaction scheme of the synthesis of lignin‐based PHUs.

Prepo‐bNH_2_ was then reacted with the lignin‐based CC, maintaining a stoichiometric ratio between NH_2_ and CC groups (Scheme 4). No catalyst was added since Prepo‐bNH_2_ already contained TBD. Acetone was used to improve the mixing of lignin‐based CC with Prepo‐bNH2. This solvent addition was particularly necessary for Lig‐CCs, which formed aggregates in the reaction media at room temperature (Figure S23). Due to the very close CC contents for all lignin‐based CC, the PHU thermosets had similar lignin contents, between 28.4 and 33.9 wt% (Table 2).

**TABLE 2:** Thermal and thermomechanical data as measured by TGA, DSC, and DMA.

PHU	Lig‐CC content, %wt	*T*_5%_, °C	*T*_d_, °C	*T*_g_, °C	*T*_α_, °C
PHU‐SW‐KL	32.6	257	373	−9 ± 1	13 ± 2
PHU‐HW‐OL	30.8	243	370	−13 ± 1	8 ± 2
PHU‐HTL‐SW‐KL	33.9	253	387	−13 ± 3	11 ± 2
PHU‐HTL‐HW‐OL	30.7	249	384	−12 ± 2	7 ± 2
PHU‐H_2_‐SW‐KL	29.7	243	381	−18 ± 1	−1 ± 2
PHU‐H_2_‐HW‐OL	28.4	251	381	−15 ± 1	3 ± 2

Abbreviations: T_5%_, temperature at 5 wt% loss in TGA. *T*_d_, temperature of the maximum degradation rate in TGA. *T*_g_, temperature at the inflexion of the DSC curve. *T*_α_, temperature at the maximum of tan δ in DMA.

**FIGURE 3:**
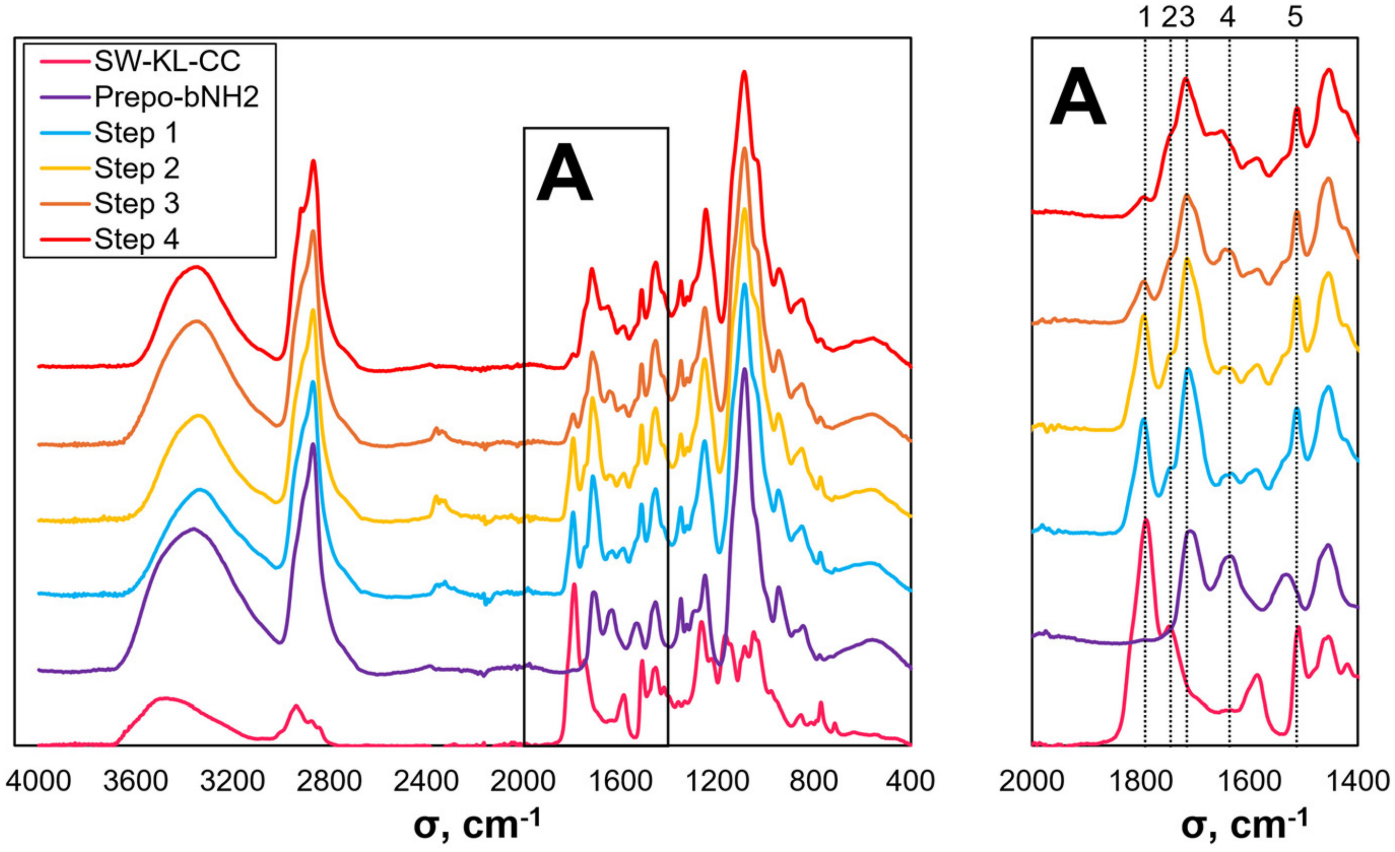
FTIR spectra of SW‐KL‐CC, Prepo‐bNH_2_, and PHU‐SW‐KL after the different curing steps. Step 1: 4 h at 80 °C. Step 2: 4 h at 120 °C. Step 3: 2 h at 160 °C under pressure. Step 4: 4 h at 160 °C. (A) Zoom of the 1400–2000 cm^−1^ region. Bands attribution: (1) C=O_CC_ band, 1790 cm^−1^. (2) Secondary C=O_CC_ band, 1740 cm^−1^. (3) C=O_urethane_ band, 1710 cm^−1^. (4) N—H_amine_ band, 1630 cm^−1^, (5) N—H_urethane_ band, 1520 cm^−1^.

A preliminary study showed that the aminolysis was relatively slow and required incremental temperature steps. This result was expected from the rather low reactivity of ether‐linked five‐membered CCs [72, 74]. In previous studies, lignin‐based CC also needed long reaction times, high temperatures (above 150 °C), and a solvent to fully react [48, 56]. The conversion of CCs would stop after a few hours at a given temperature. A widely documented explanation of this phenomenon is that H‐bonds limit the reactant mobility as the aminolysis progresses [75, 77]. A low mobility could also limit the reactivity of β‐hydroxyamines, despite previous reports showing their excellent reactivity toward CCs [78]. Finally, the PHUs were synthesized at 80 °C for 4 h, then at 120 °C for 4 h, and finally at 160 °C for 6 h (Scheme 4). This incremental process led to the progressive disappearance of CC and amine bands at 1790 and 1630 cm^−1^ in FTIR, as seen in Figure 3 for PHU‐SW‐KL‐CC. Lig‐CCs and oLig‐CCs exhibited a similar reactivity, as seen from the FTIR spectra measured after each curing step (Figures S24, S25), meaning that lignin molar mass is not a key factor to explain the low reactivity [17]. At the end of the final curing step, the conversion is almost complete, with only small amounts of remaining CC groups detected by FTIR (Figures 3 and S26).

### 3.4 PHUs Characterization

The PHUs were analyzed through TGA, DSC, DMA, swelling tests, and tensile tests to assess the effect of the (i) lignin type, (ii) the depolymerization methodology, and (iii) the functionality of the lignin‐based CC on the material properties.

Despite the different structures of the feedstocks, all PHUs displayed similar thermal degradation behaviors (Table 2 and Figures S27, S28), with *T*_5%_ above 250 °C, *T*_d_ between 370 and 380 °C, and residues at 700 °C between 11 and 18 wt%. The main *T*_d_ of about 380 °C is associated with the degradation of ether and urethane bonds in the network [47]. All the materials exhibited *T*_g_ below room temperature, between −9 and −18 °C (Table 2 and Figure S29). The molar mass and *T*_g_ of the Lig‐CCs and oLig‐CCs only have a minor influence on the *T*_g_ of the PHUs.

**FIGURE 4:**
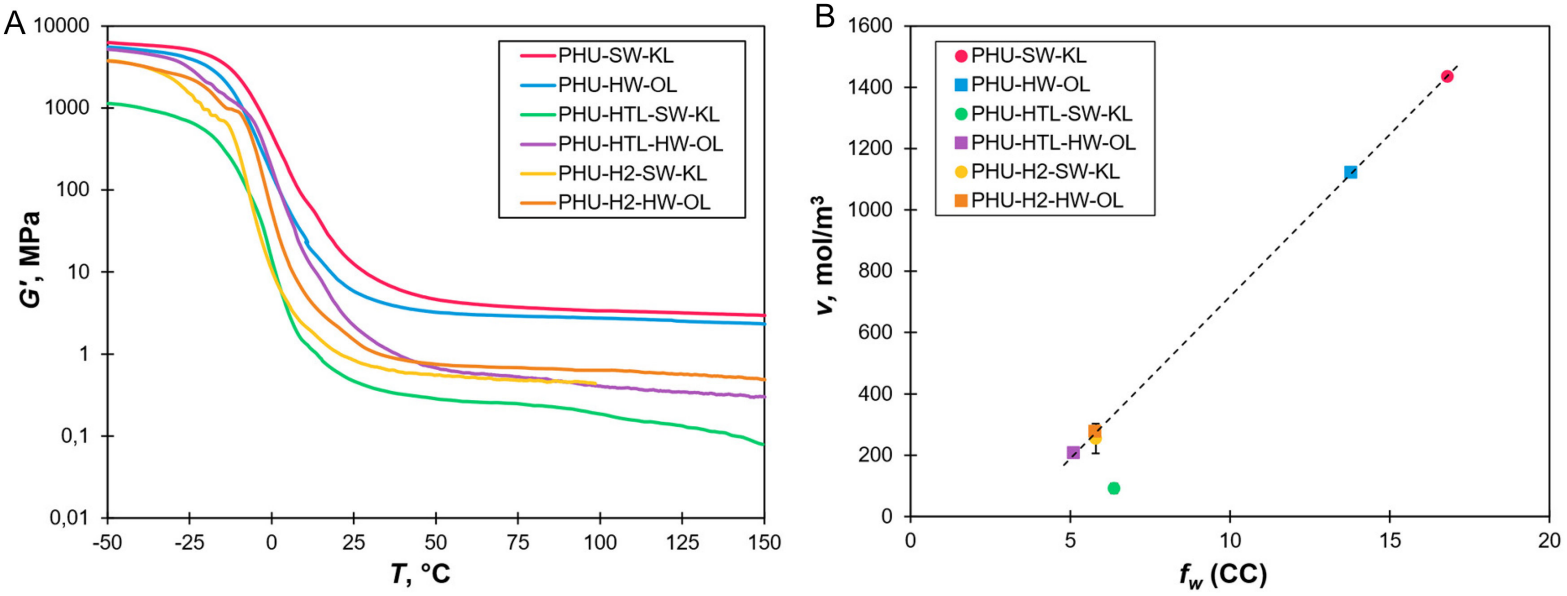
(A) Storage modulus (*G′*) measured by DMA. (B) Crosslink density (*ν*) of the PHUs depending on the average functionality of Lig‐CCs and oLig‐CCs.

DMA was used to study the thermomechanical behavior of the materials (Figures 4 and S30). *T*_α_ was found to be well correlated with the *T*_g_ measured by DSC (Table 2 and Figure S31). All the materials showed a clear rubbery plateau after *T*_α_, confirming their crosslinked nature (Figure 4A). The crosslink density was calculated from the value of the storage modulus *Gʹ* at *T*_α_ + 50 °C, according to the rubber elasticity theory (Equation (4)):

(4) $$\upsilon \left(\mathrm{mol}\;m^{-3}\right)=\frac{G'(T_{\alpha }+50)}{R(T_{\alpha }+50)}$$

It was found to be directly correlated to the functionality of the Lig‐CCs or oLig‐CCs precursors (Figure 4B). Indeed, lignin brings the crosslinking points in the networks, and since the lignin content is similar for all the studied PHUs, the functionality of the lignin precursors directly controls the material crosslink density. Only the crosslink density of PHU‐HTL‐SW‐KL was found to be unexpectedly low (Figure 4B), which could be explained by a higher proportion of network defects, such as loops or dangling ends, compared to the other materials [79]. Further analyses would be needed to better understand this peculiar behavior.

**FIGURE 5:**
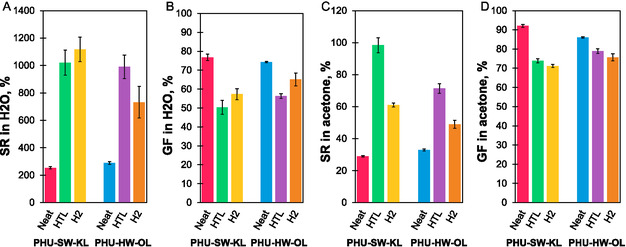
Results of swelling tests of the PHUs. (A) Swelling ratio and (B) gel fraction in water. (C) Swelling ratio and (D) gel fraction in acetone.

SR and GF of the PHUs were measured to assess the crosslink densities through another approach (Figure 5 and Table S8). The swelling tests were performed in water and acetone. All PHUs have a higher affinity toward water than toward acetone, which can be explained by the large quantity of H‐bond donors in their chemical structure. PHUs prepared from oLig‐CCs have significantly higher SR and lower GF than those prepared with Lig‐CCs. It is related to their lower functionality, which in turn results in reduced crosslinking. SR and GF are quite well correlated with the crosslink density measured by DMA (Figure S32). The PHUs have a relatively low GF, as low as 50% in water for PHU‐HTL‐SW‐KL. The soluble fraction was analyzed by ^1^H NMR, showing that it was mainly composed of some unreacted prepolymer and CC, with very few urethane bonds (Figure S33).

**FIGURE 6:**
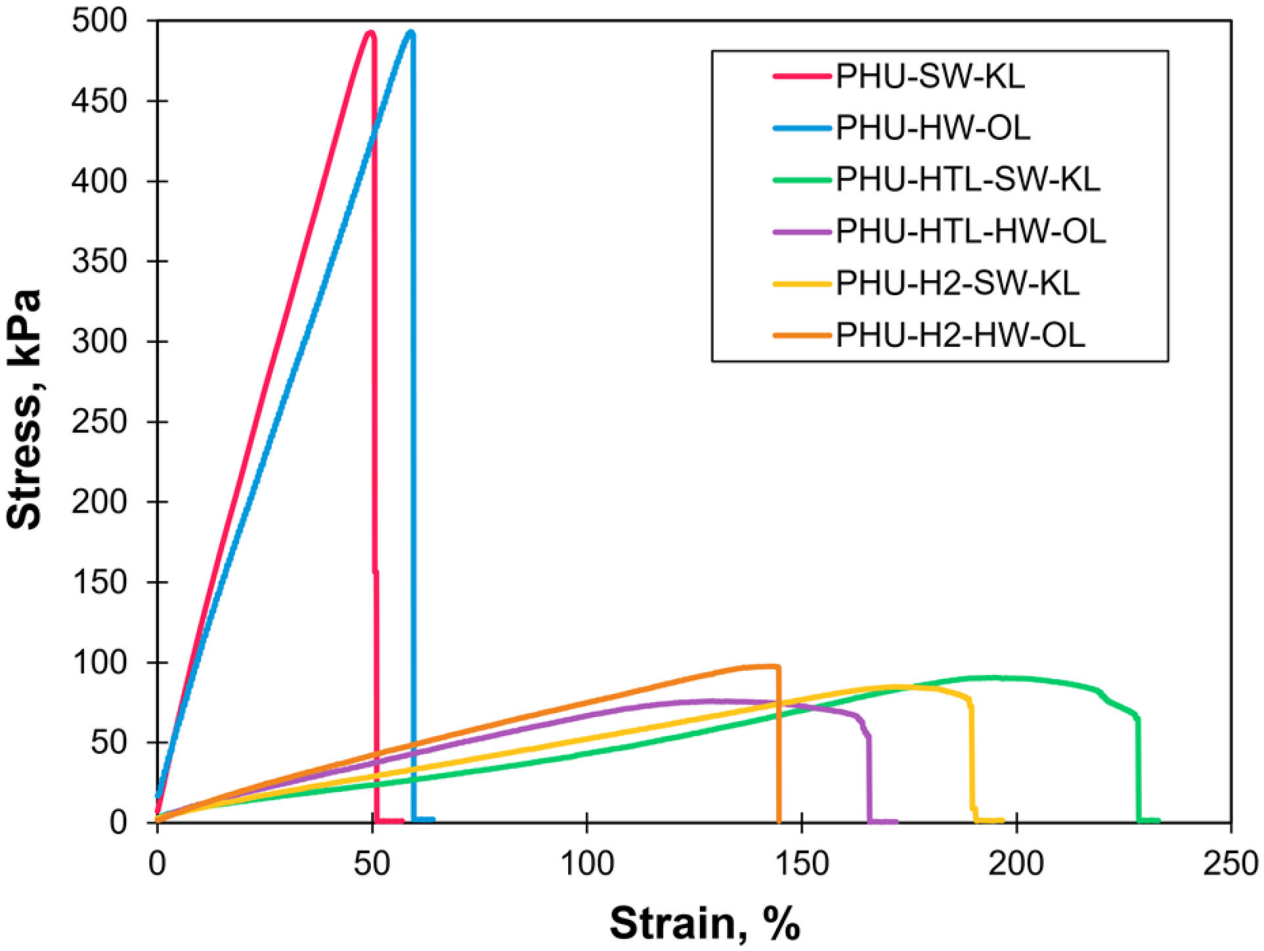
Representative stress–strain curves measured by uniaxial tensile tests.

The mechanical properties of materials were measured by uniaxial tensile tests at room temperature, above *T*_g_ for all materials (Figures 6 and S34 and Table S9). PHUs from oLigs were particularly soft and flexible, with low Young's modulus between 50 and 80 kPa and elongations up to 220%. In comparison, Ligs led to materials with higher rigidity. PHU‐SW‐KL and PHU‐HW‐OL displayed higher modulus of 0.9 ± 0.05 and 1.4 ± 0.3 MPa, and lower elongations at break of 46 ± 9 and 63 ± 2%, respectively.

**FIGURE 7:**
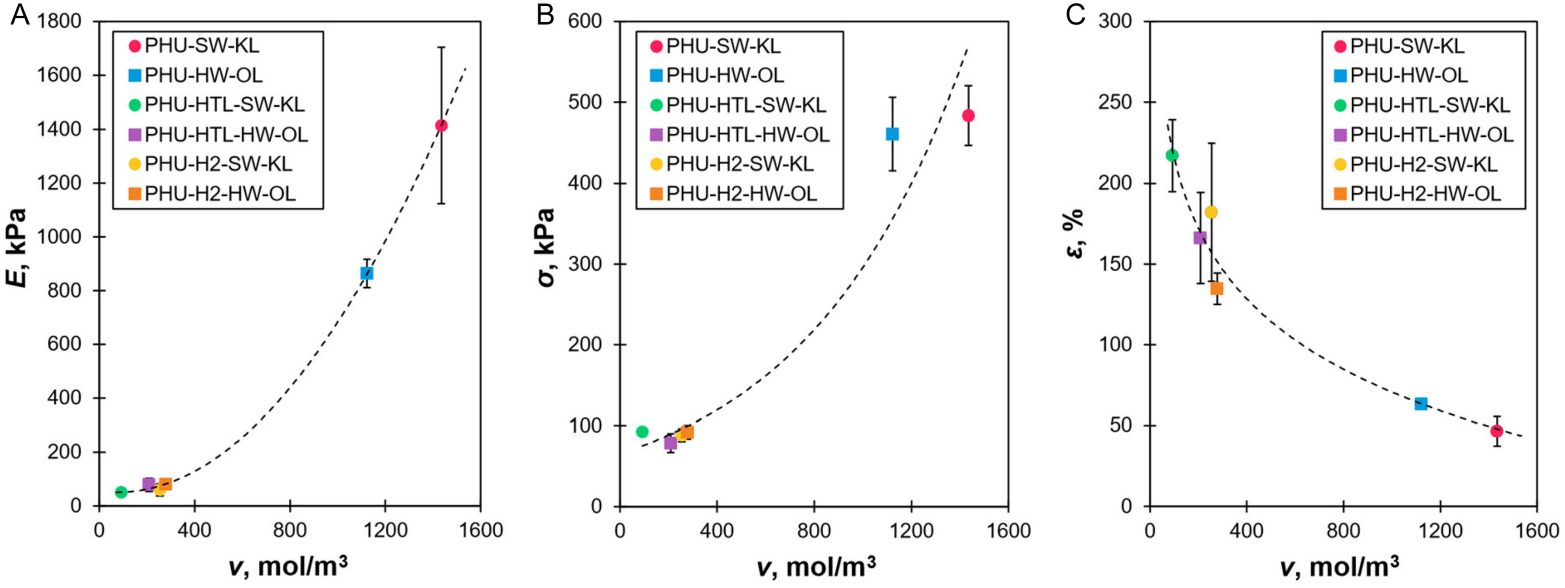
Correlation between the mechanical properties measured by uniaxial tensile tests and the crosslink density measured by DMA. (A) Young's modulus, (B) tensile strength, and (C) elongation at break.

The mechanical properties of the materials were found to be correlated with the crosslink density (Figure 7). Although this is expected for crosslinked polymer networks, it is noteworthy that other factors, such as the lignin type, structure, molar mass, or functional group content, appear to have a negligible influence. Despite substantial structural differences among the tested Ligs and oLigs, the crosslink density is mainly controlled by the functionality (Figure 4B), which in turn emerges as the key parameter governing the mechanical properties of the lignin‐based PHUs. This observation is critical to understand the “structure–‍properties” relationships in lignin‐based materials and should be further validated in other kinds of lignin‐based polymer materials.

**FIGURE 8:**
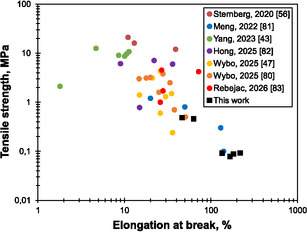
Comparison of lignin‐based NIPUs tensile properties reported in the literature. Each color represents one study with the corresponding tested materials.

Significant differences can be observed between the mechanical data of the PHUs presented in this study and previously reported lignin‐based NIPUs (Figure 8) [43, 47, 56, 80, 83]. For instance, PHUs from oLigs present the highest elongations ever reported with lignin‐based NIPUs. However, a fair comparison of NIPU properties is often difficult due to the diversity of structures, depending on the choice of lignin, choice of soft segment, functionalization method, functionality of monomers, and lignin content. When comparing at fixed lignin contents, a clear proximity arises with the materials reported by Meng et al. [81], which were also produced with low molar mass lignins (Figure S35). Both studies led to flexible PHUs at relatively high lignin contents, which is essential for elastomer applications. Overall, depolymerization of lignins allows the production of lignin‐based PHUs with higher flexibility, lower crosslinking densities, and similar thermal stabilities.

## 4 Conclusion

This study demonstrates the successful synthesis of renewable and safe aromatic PHUs from different technical lignins and lignin oligomers obtained by partial depolymerization. Six samples displaying varied backbone structures, functionalities, and molar masses were obtained from two different types of lignins (Kraft and organosolv), and two depolymerization methodologies (hydrothermal and reductive). They were functionalized to lignin‐based CC building blocks through an optimized one‐pot methodology, with substantial improvements in the green metrics, and polymerized with diamines to yield PHUs with high lignin contents.

All lignin‐based CC displayed a similar reactivity toward aminolysis. The crosslinking density of PHUs, and consequently their mechanical properties, were directly correlated with the functionality of lignin‐based CCs. Significantly different mechanical behaviors can thus be achieved with lignin‐based CCs of varying functionalities, offering opportunities for a variety of applications in which soft or more rigid materials are required.

Overall, this study provides a better understanding of the key factors controlling the properties of lignin‐based materials. Despite the very different structures among the six selected lignin samples, the material properties were mainly affected by the functionality of the lignin‐based building blocks. The lignin type or depolymerization process, which strongly influence the lignin structure, does not significantly influence the material properties if the same functionality is reached. This important result lays the foundation for a better understanding of “structure–properties” relationships in lignin‐based materials, a key to improve the design of high‐performance aromatic networks. It should now be extended to other types of lignin‐based polymer materials, but it also points to the need for new methods to accurately assess lignin functionality distributions, as the approach used here relies on SEC data that can be biased by the standard calibration.

## Author Contributions

**Nathan Wybo**: investigation, data curation, conceptualization, methodology, visualization, writing – original draft, writing – review and editing. **Hugo Lilti**: investigation (depolymerization), methodology, writing – review and editing. **Dorothée Laurenti**: supervision, funding acquisition, writing – review and editing. **Christophe Geantet**: supervision, funding acquisition. **Antoine Duval**: conceptualization, data curation, supervision, funding acquisition, writing – review and editing. **Luc** **Avérous**: funding acquisition, supervision, writing – review and editing. All authors have approved the final version of the manuscript.

## Funding

This study was supported by Agence Nationale de la Recherche (ANR‐21‐CE43‐0026).

## Conflicts of Interest

The authors declare the following financial interests/personal relationships, which may be considered as potential competing interests: Luc Averous and Dorothée Laurenti report that financial support was provided by French National Research Agency. Administrative support and equipment, drugs, or supplies were provided by CNRS Délégation Centre‐Est. Article publishing charges and equipment, drugs, or supplies were provided by University of Strasbourg and IRCELyon. If there are other authors, they declare that they have no known competing financial interests or personal relationships that could have appeared to influence the work reported in this article.

## Supporting information

Supplementary Material

## Data Availability

The data supporting this article have been included as part of the Supporting Information. Additional references were cited in the Supporting Information [43, 47, 48, 53, 55, 57, 80, 82, 84].
