# Supplementary material for: Unraveling the Role of Lignin Oligomers Functionality for the Synthesis of Nonisocyanate Polyurethanes
Source: ChemSusChem. 2026 Aug 7;19(15):e70953. doi: 10.1002/cssc.70953 (PMC13450534; doi:10.1002/cssc.70953)
Supplement: Supplementary file 1 [file CSSC-19-e70953-s001.pdf]

Unraveling the Role of Lignin Oligomers Functionality for the Synthesis of Non-isocyanate Polyurethanes

Nathan Wybo[1], Hugo Lilti[2], Dorothée Laurenti[2], Christophe Geantet[2], Antoine Duval[1,3,], Luc Avérous[1,]**

1. BioTeam/ICPEES-ECPM, UMR 7515, CNRS Université de Strasbourg, 25 rue Becquerel, 67087 Strasbourg Cedex 2, France

2. IRCELYON, UMR 5256, CNRS Université Lyon 1 UCBL, 2 avenue Albert Einstein, 69626 Villeurbanne Cedex

3. Soprema, 15 rue de Saint Nazaire, 67100 Strasbourg, France

Corresponding authors: *antoine.duval@unistra.fr; *luc.averous@unistra.fr

Supplementary Information

Contents

LIGNINS DEPOLYMERIZATION 3

- FTIR, ^{1}H AND ^{31}P NMR CHARACTERIZATION OF LIGS AND OLIGS 3
- SEC CHARACTERIZATION OF LIGS AND OLIGS 5
- ^{1}H-^{13}C HSQC CHARACTERIZATION OF LIGS AND OLIGS 5
- TGA AND DSC CHARACTERIZATION OF LIGS AND OLIGS 10

LIGNINS FUNCTIONALIZATION TO POLYCYCLOCARBONATES 12

- GREEN METRICS OF CC FUNCTIONALIZATION 12
- FTIR, ^{1}H NMR, ^{31}P NMR, AND ^{13}C NMR CHARACTERIZATION OF LIG-CCS AND OLIG-CCS 13
- MASS YIELD AND CC YIELD 17
- SEC, TGA, AND DSC CHARACTERIZATION OF LIG-CCS AND OLIG-CCS 19

PHUS SYNTHESIS 22

- ^{1}H NMR OF PEG DERIVATIVES 22
- QUANTITATIVE NMR FOR THE EVALUATION OF CC AND NH_2 GROUPS IN PEG DERIVATIVES 22
- PHU REACTION MIXTURE MIXING 23
- FTIR ALONG PHUS SYNTHESIS 25

PHUS CHARACTERIZATION 28

- THERMAL AND MECHANICAL PROPERTIES BY TGA, DSC, AND DMA 28
- SWELLING PROPERTIES 30
- TENSILE PROPERTIES 31
- MECHANICAL PROPERTIES COMPARISON WITH LITERATURE 33

SUPPLEMENTARY INFORMATION REFERENCES 34

Lignins depolymerization

FTIR, ^{1}H and ^{31}P NMR characterization of Ligs and oLigs

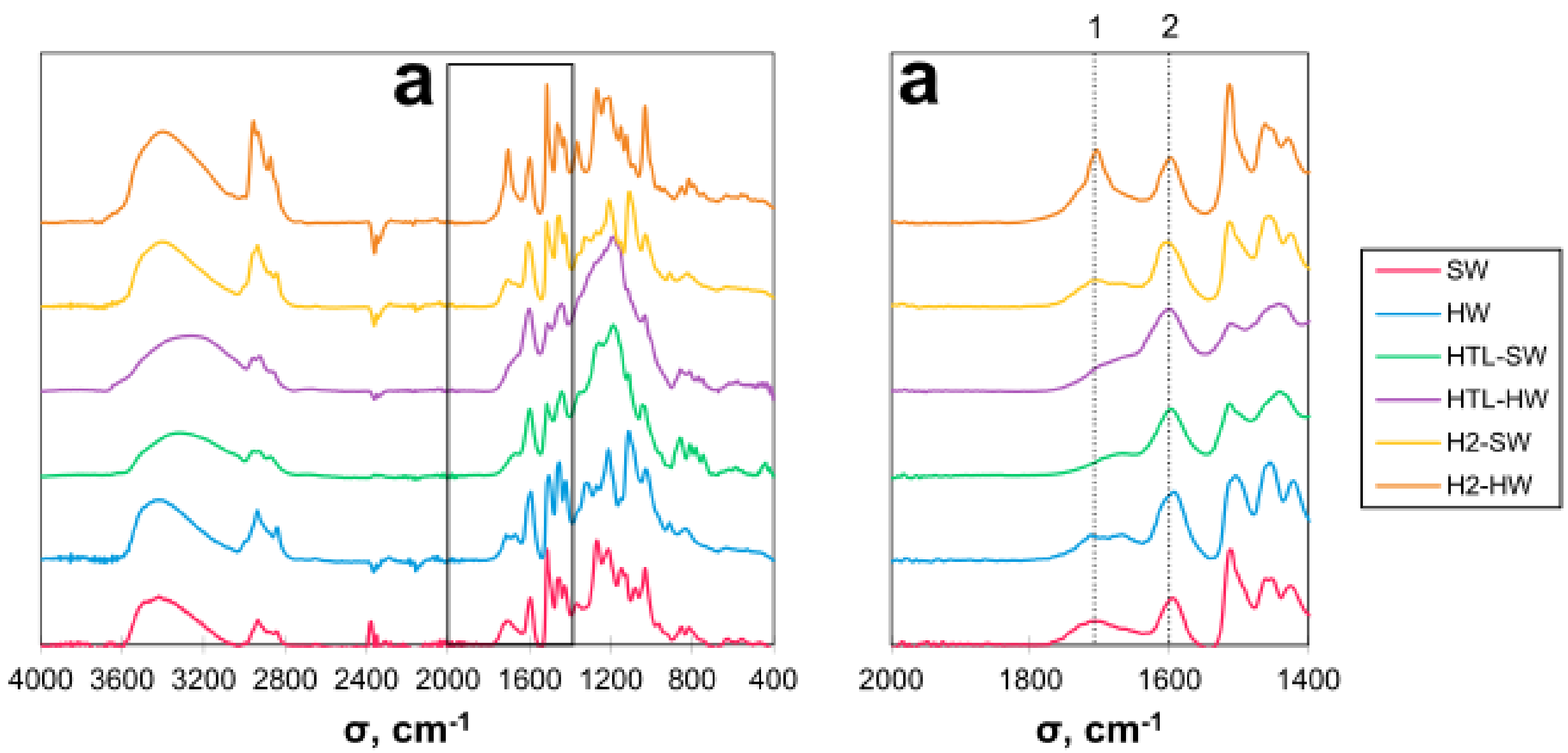

Figure S1. FTIR spectra of Ligs and oLigs. (a) zoom on the 1400-2000 cm^{-1} region. 1) C=O band, 1700 cm^{-1} (residual carboxylic acids, ketones, and aldehydes). 2) C=C band, 1590 cm^{-1}.

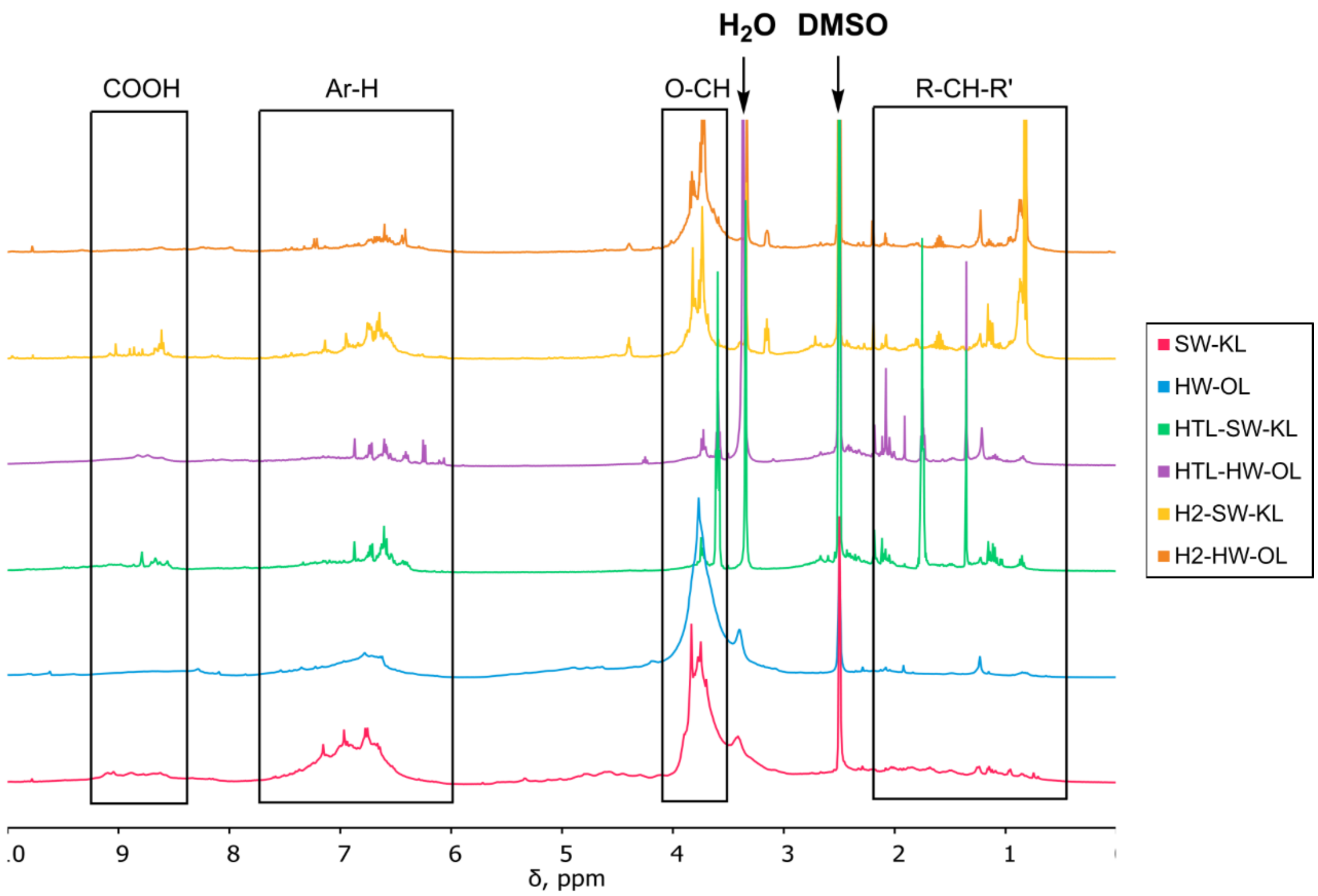

Figure S2. ^{1}H NMR spectra of Ligs and oLigs. Solvent: DMSO-*d₆*.

Table S1. Quantification of hydroxyl content of Ligs and oLigs as measured by quantitative ^{31}P NMR. AlOH: aliphatic alcohol. PhOH: phenols. COOH: carboxylic acids. PhOH content includes catechols and gallols. Catechols and Gallols quantifications are only indicative.

	**AlOH, mmol/g**	**PhOH, mmol/g**	*catechols, mmol/g*	*gallols, mmol/g*	**COOH, mmol/g**	**Total OH, mmol/g**
**SW-KL**	2.03	3.94	-	-	0.36	6.33
**HW-OL**	1.80	2.77	-	-	0.11	4.68
**HTL-SW-KL**	0.09	8.07	4.0	0.7	0.20	8.36
**HTL-HW-OL**	0.12	7.56	2.5	1.8	0.17	7.85
**H_2-SW-KL**	1.39	5.01	-	-	0.22	6.62
**H_2-HW-OL**	0.98	4.11	-	-	0.08	5.17

SEC characterization of Ligs and oLigs

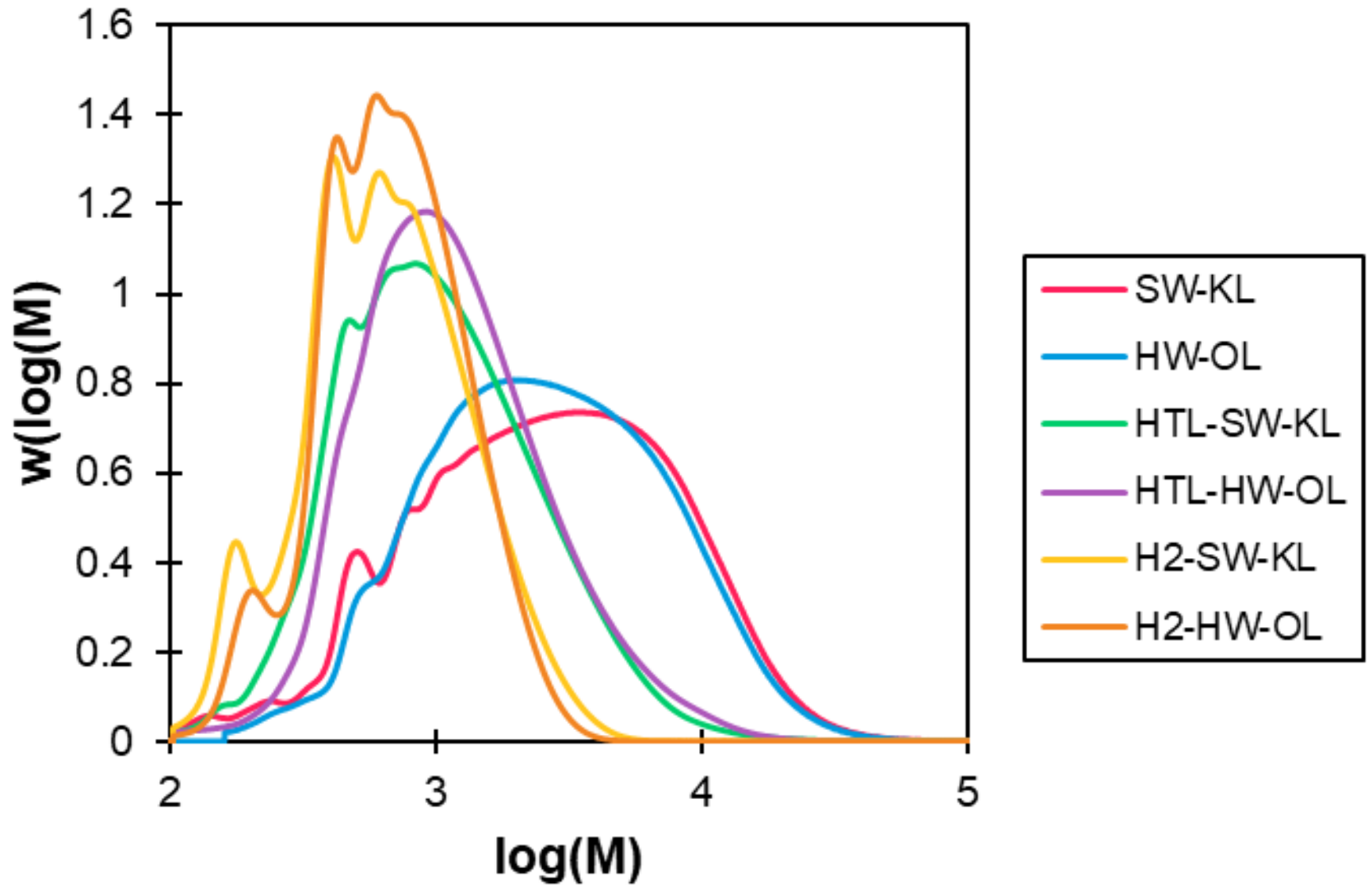

Figure S3. SEC chromatograms for acetylated Ligs and oLigs normalized on total signal. Detector: UV transmission. Solvent: THF. Standards: PS.

Table S2. SEC data for Ligs and oLigs.

	M_n, g/mol	M_w, g/mol	*Đ*
**SW-KL**	1347	4399	3.3
**HW-OL**	1582	4196	2.7
**HTL-SW-KL**	737	1510	2.0
**HTL-HW-OL**	861	1668	1.9
**H_2-SW-KL**	507	838	1.7
**H_2-HW-OL**	561	820	1.5

^{1}H-^{13}C HSQC characterization of Ligs and oLigs

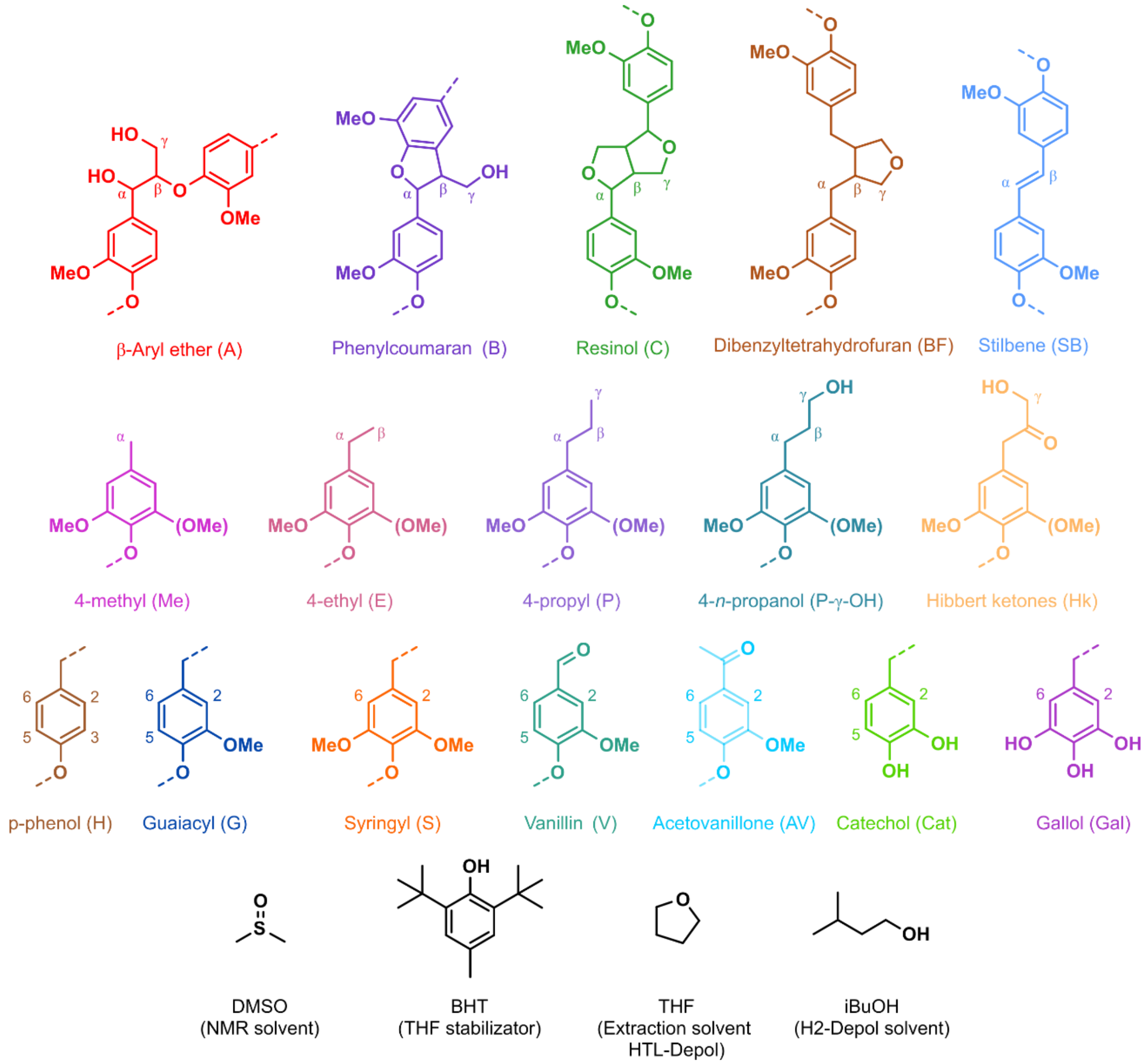

Figure S4. Structures attribution for ^{1}H-^{13}C HSQC according to Smit et al.[1]

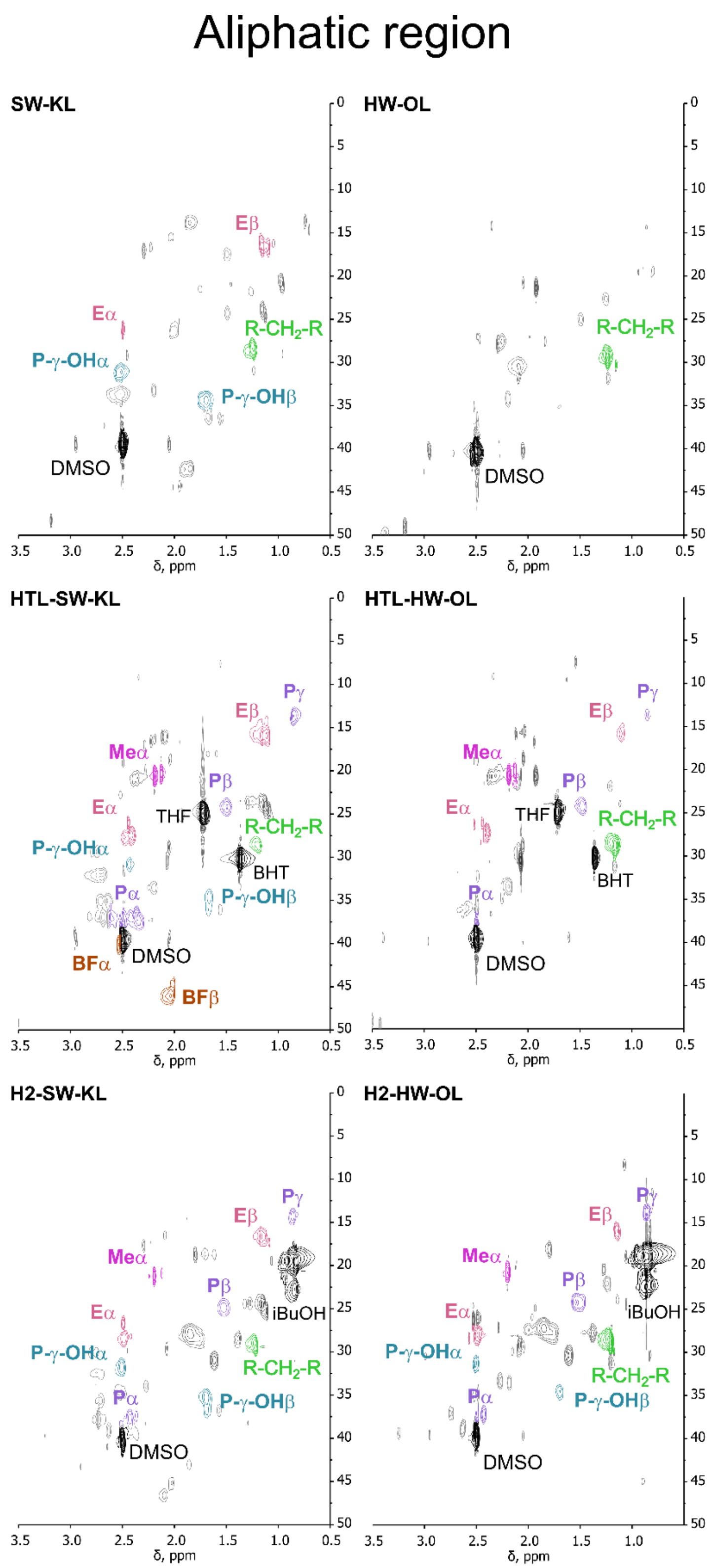

Figure S5. ^{1}H-^{13}C HSQC of Ligs and oLigs, aliphatic region. Solvent: DMSO-d_6

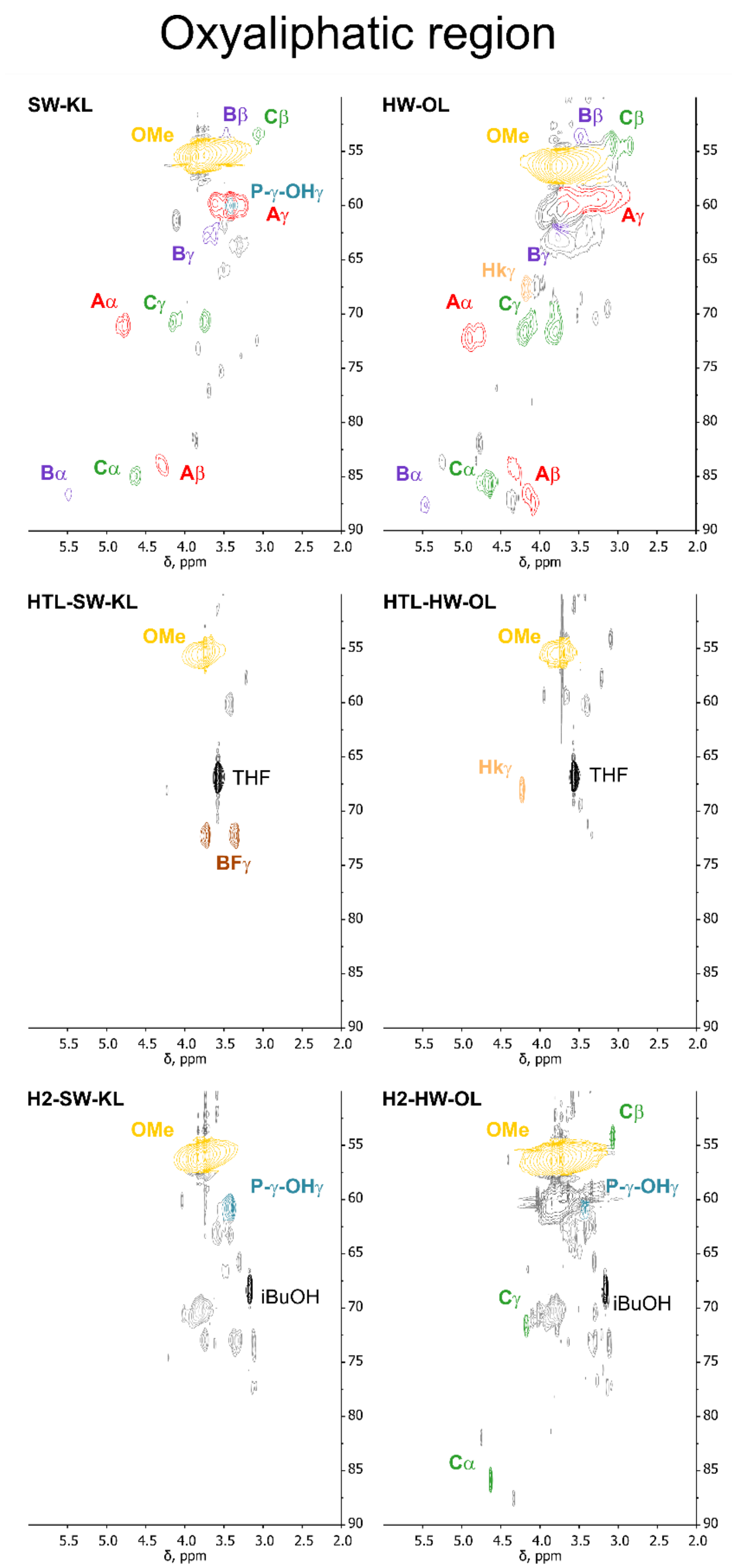

Figure S6. ^{1}H-^{13}C HSQC of Ligs and oLigs, oxyaliphatic region. Solvent: DMSO-d_6

Aromatic region

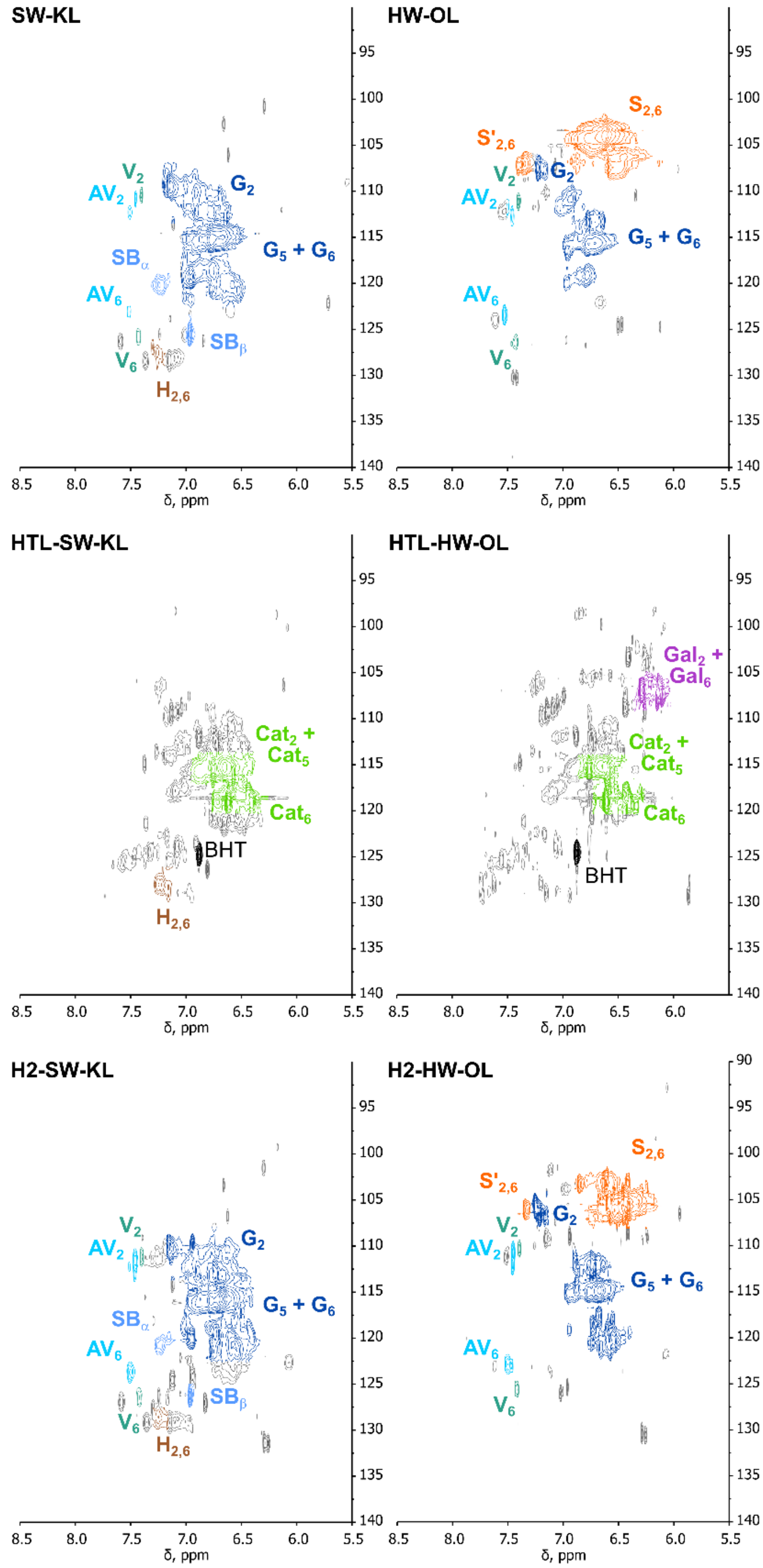

Figure S7. ^{1}H-^{13}C HSQC of Ligs and oLigs, aromatic region. Solvent: DMSO-d_6

TGA and DSC characterization of Ligs and oLigs

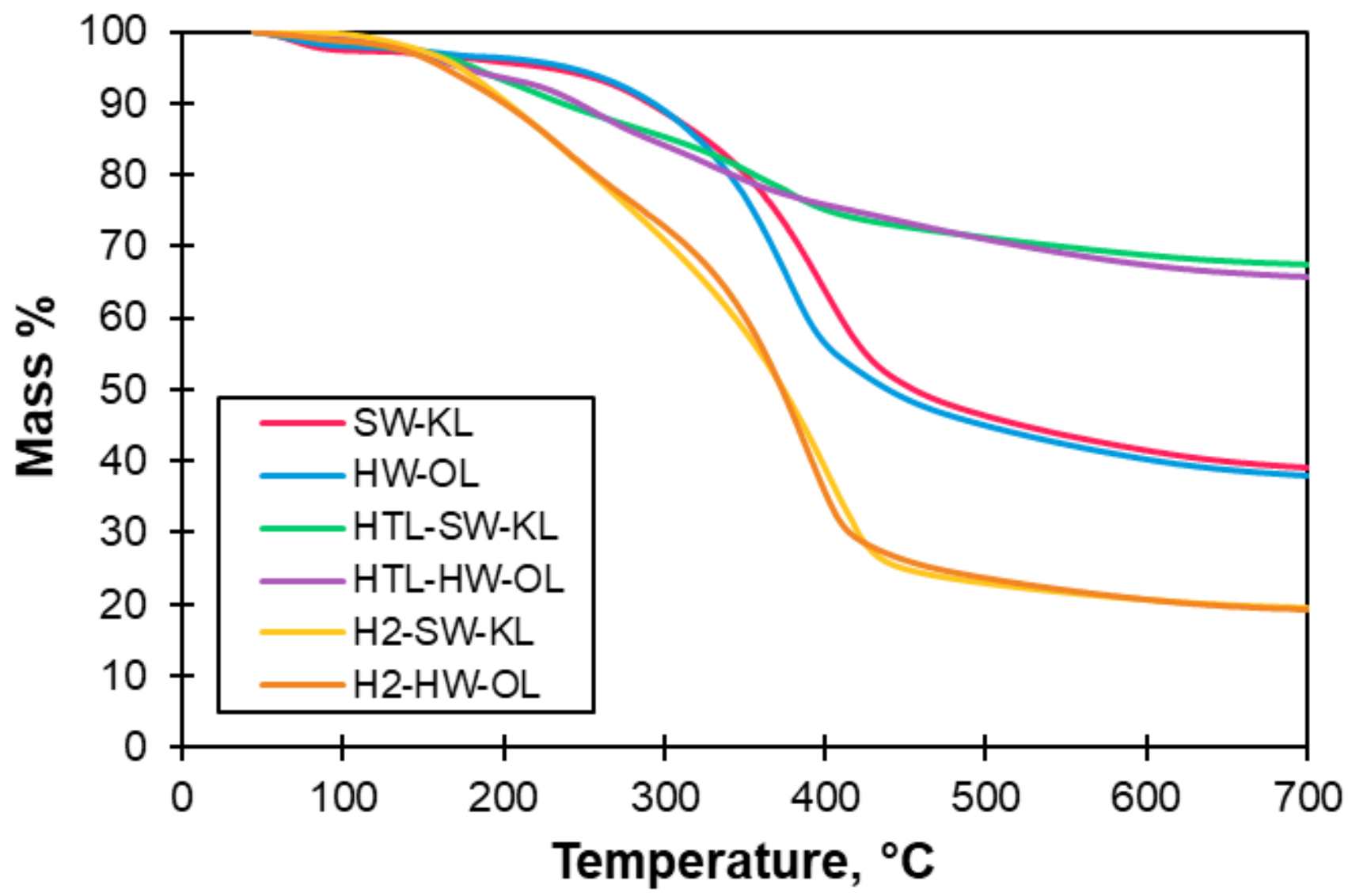

Figure S8. TGA curve for Ligs and oLigs. Temperature ramp: 20 °C/min. Gas: N_2.

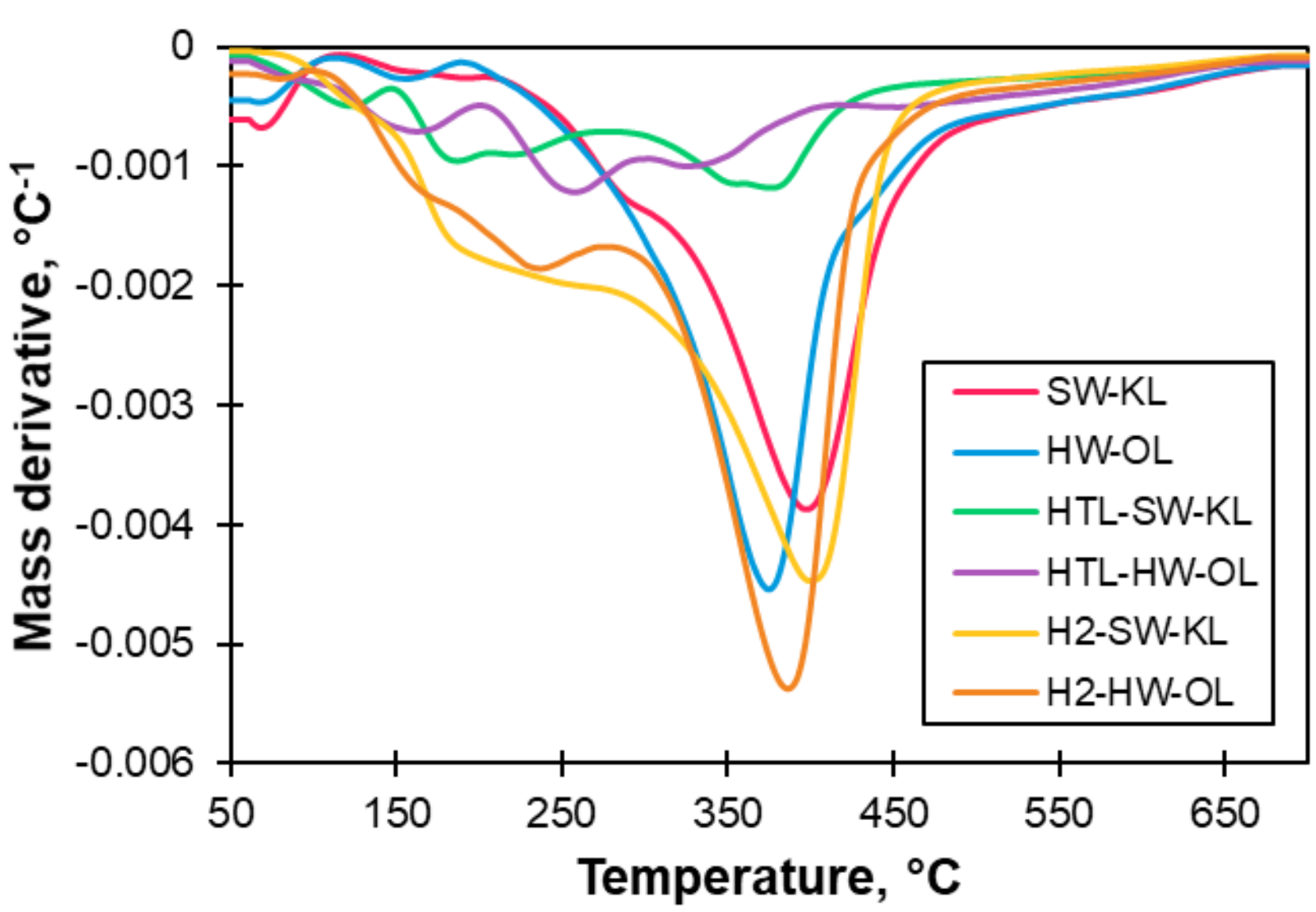

Figure S9. DTG curve for Ligs and oLigs. Temperature ramp: 20 °C/min. Gas: N_2.

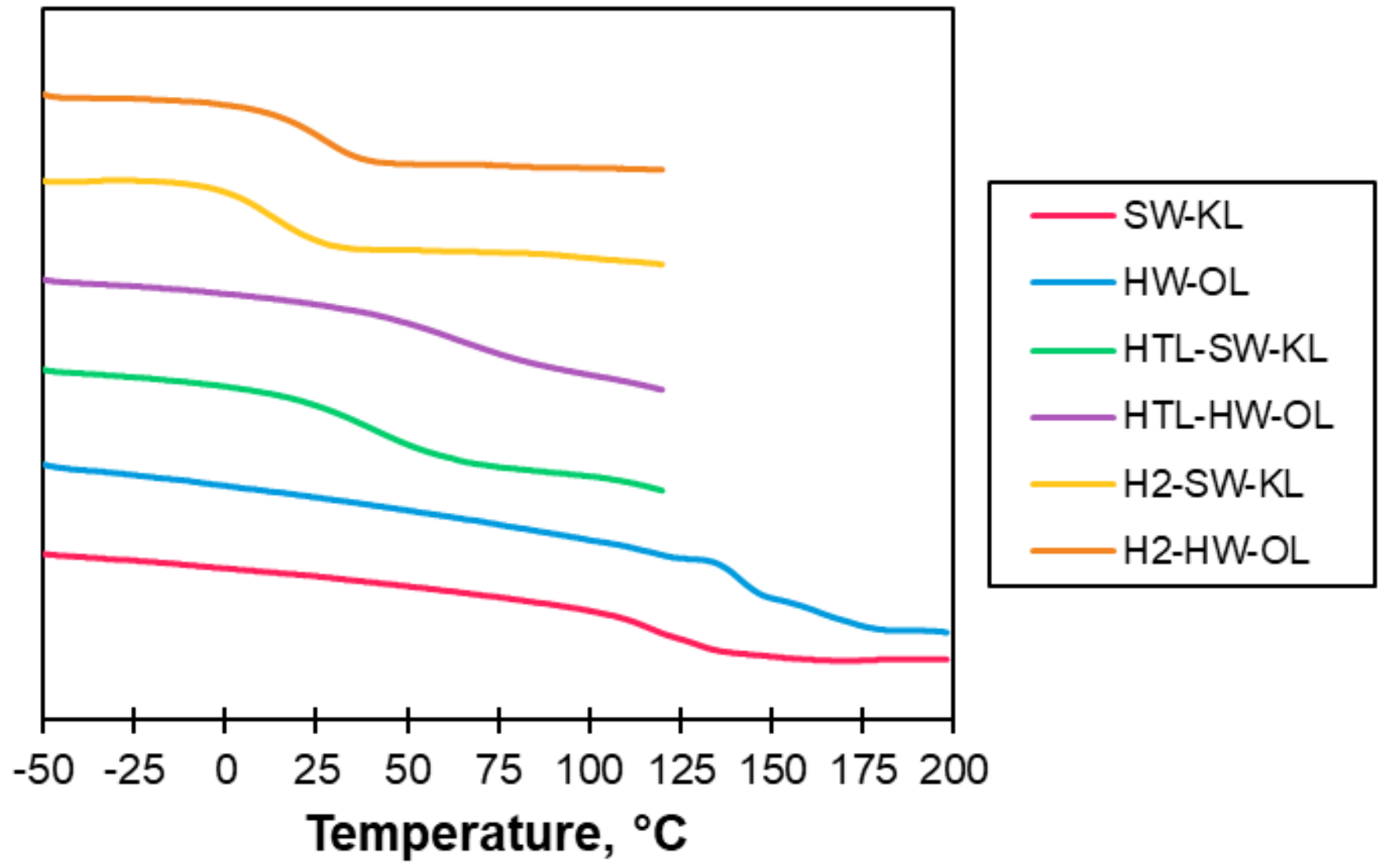

Figure S10. DSC curve for Ligs and oLigs. Temperature ramp: 10 °C/min. For oLigs, temperatures above 120 °C led to significant degassing.

Lignins functionalization to polycyclocarbonates

Green metrics of CC functionalization

Table S3. Green metrics comparison between the functionalization methodologies for the production of Lig-CCs.

	Mass yield	E factor[a]	E factor[b]	Temperature and duration	Solvents	CMR or toxic chemicals
**ECH+CO_2 Two steps[2–4]**	63%[2]	130[2]	≫ 500[2]	1) 70 °C, 3 h 2) 80 °C, 20 h[2]	Acetone[2] (or DMF[3]/ DMAc[4])	ECH
**GC+DMC Two steps[5,6]**	106%[6]	13[5]	> 500[5]	1) 150 °C, 1.5 h 2) 75 °C, 4 h[6]	DMSO	DBU
**GC+DMC One-pot** (HW-OL)	149%	5	60	130 °C, 6 h + 90 °C, 4 h	/	/

[a] organic chemicals only. [b] with water and salts.

FTIR, ^{1}H NMR, ^{31}P NMR, and ^{13}C NMR characterization of Lig-CCs and oLig-CCs

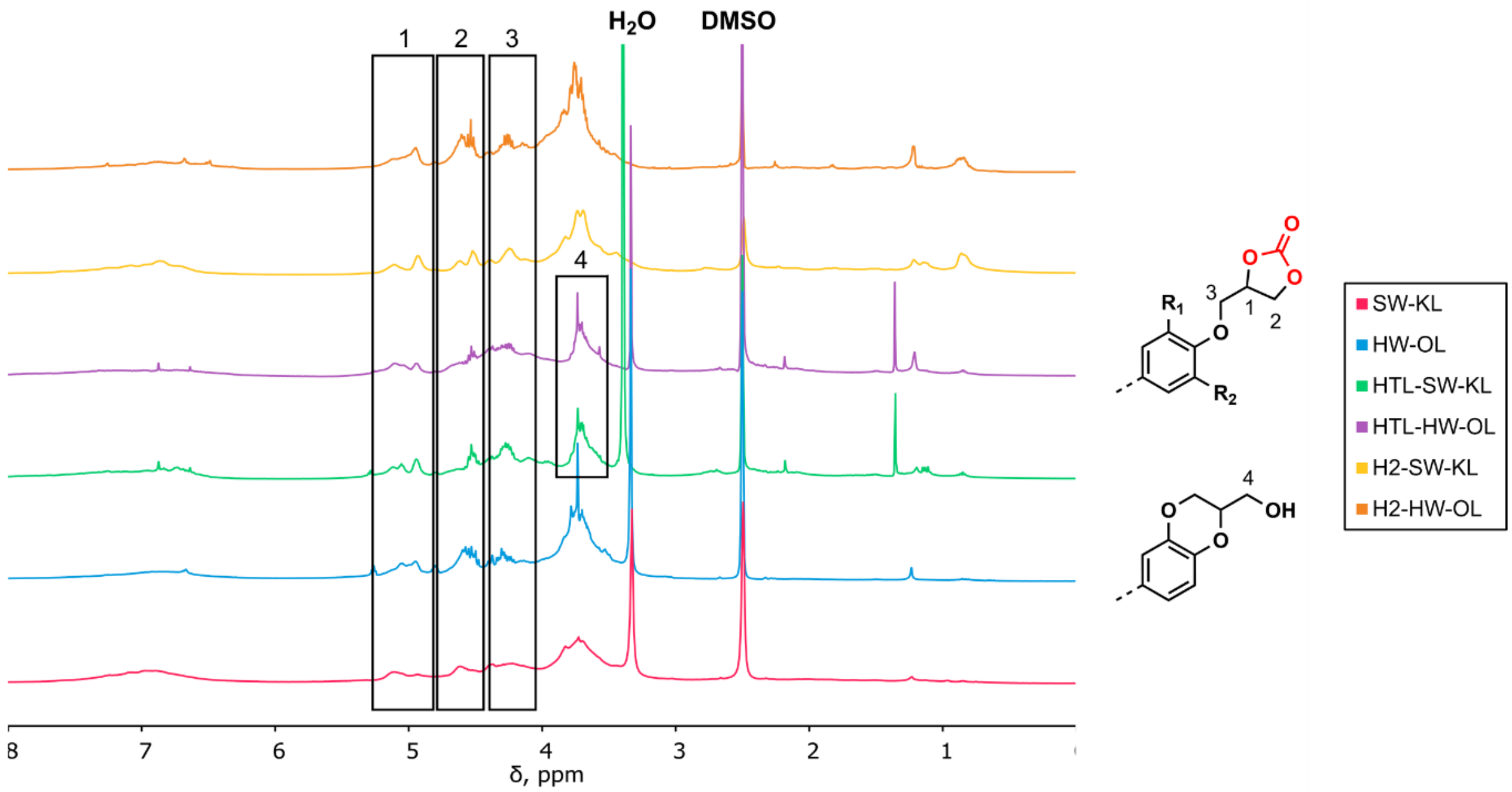

Figure S11. ^{1}H NMR spectra of Lig-CCs and oLig-CCs. Solvent: DMSO-d_6.

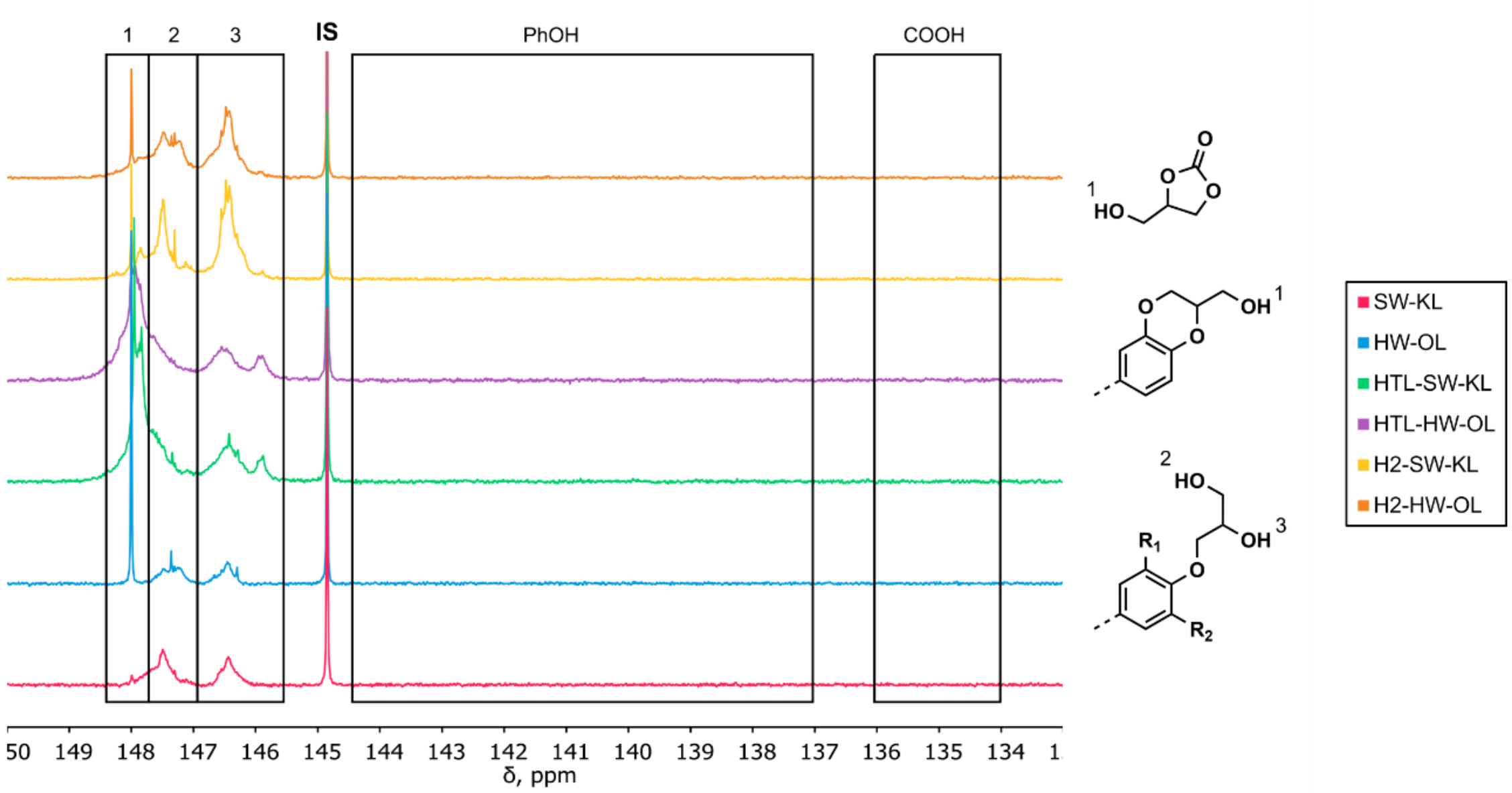

Figure S12. Quantitative ^{31}P NMR spectra of Ligs and oLigs and structure attribution. AlOH: aliphatic alcohol. PhOH: phenols. COOH: carboxylic acids. IS: internal standard, cholesterol. Solvent: $CDCl_3$/Pyridine 1/1.6.

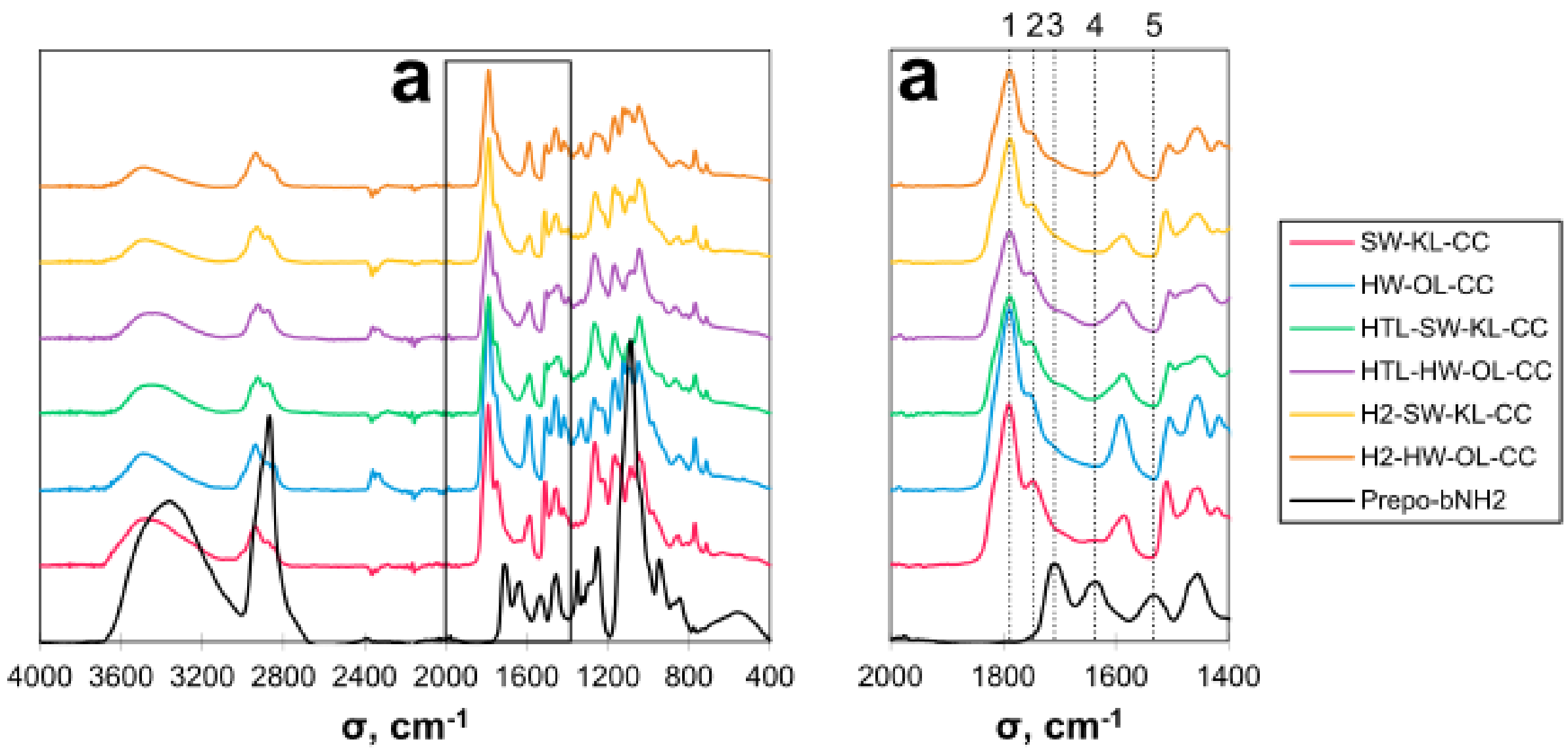

Figure S13. FTIR spectra of Lig-CCs, oLig-CCs, and Prepo-bNH_2. 1) $C{=}O_{CC}$ band, 1790 cm^{-1}. 2) Secondary $C{=}O_{CC}$ band, 1740 cm^{-1}. 3) $C{=}O_{urethane}$ band, 1710 cm^{-1}. 4) $N\text{-}H_{amine}$ band, 1630 cm^{-1}, 5) $N\text{-}H_{urethane}$ band, 1520 cm^{-1}.

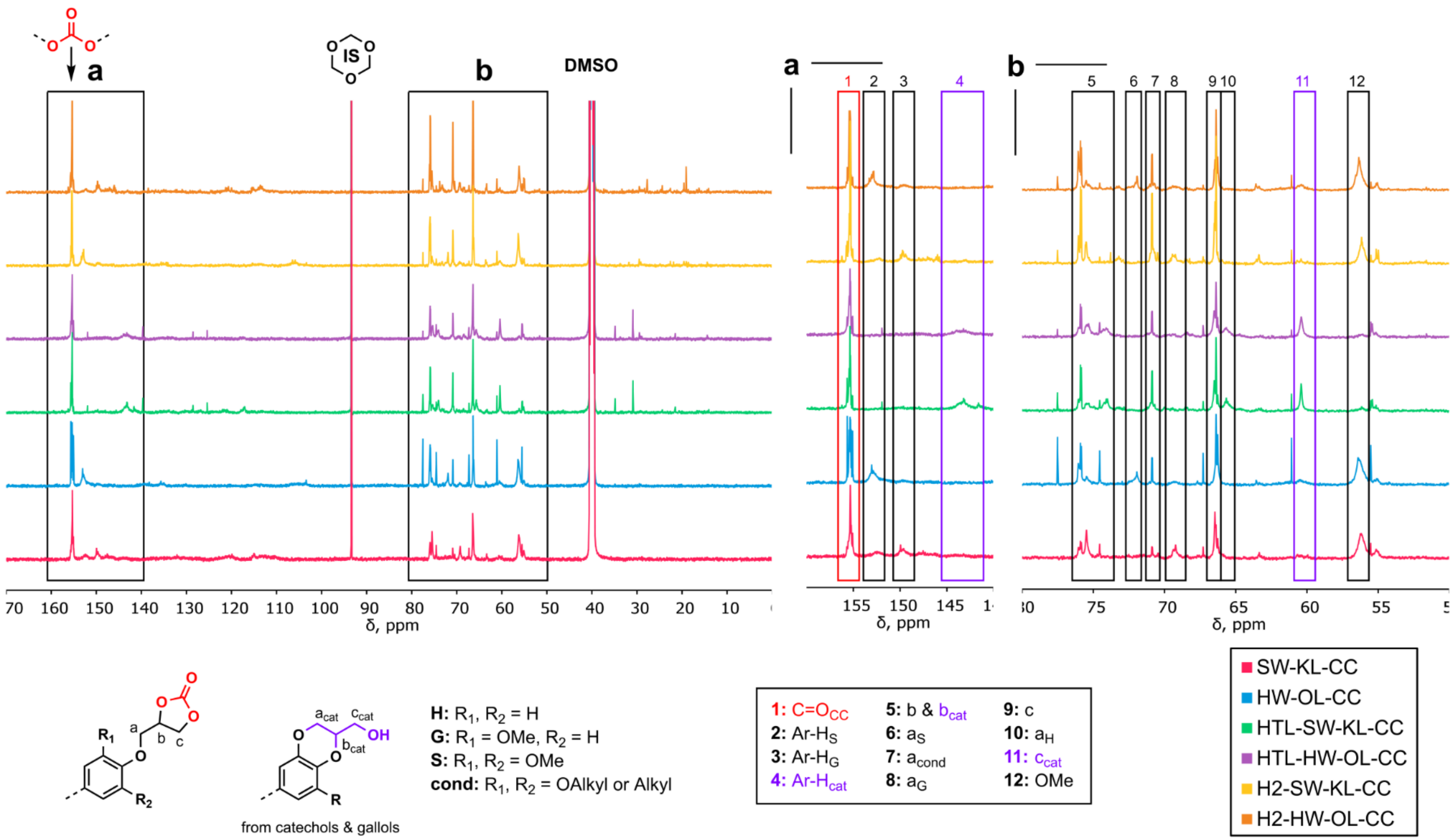

Figure S14. Quantitative ^{13}C NMR spectra of Lig-CCs and oLig-CCs in DMSO-d_6 using 1,3,5-trioxane as an internal standard (IS). (a) zoom on the region 140-160 ppm. (b) zoom on the region 50-80 ppm.

Table S4. Quantification of functional groups of Lig-CCs and oLig-CCs as measured by quantitative ^{31}P NMR and quantitative ^{13}C NMR. AlOH: aliphatic alcohol. PhOH: phenols. COOH: carboxylic acids. CC: cyclocarbonates. f_w (CC): weight-average functionality in CC groups.

	**AlOH, mmol/g**	**PhOH, mmol/g**	**COOH, mmol/g**	**Total OH, mmol/g**	**CC, mmol/g**	f_w **(CC)**
**SW-KL-CC**	0.58	0.00	0.00	0.58	2.8	17
**HW-OL-CC**	0.36	0.00	0.00	0.36	2.8	14
**HTL-SW-KL-CC**	2.36	0.00	0.00	2.36	2.7	5.1
**HTL-HW-OL-CC**	2.30	0.00	0.00	2.30	3.1	6.4
**H_2-SW-KL-CC**	2.31	0.00	0.00	2.31	3.5	5.8
**H_2-HW-OL-CC**	1.94	0.00	0.00	1.94	3.3	5.8

Mass yield and CC yield

Table S5. Expected mass yields* and CC contents of Lig-CCs and oLig-CCss if all phenols were converted to CC. Comparison with actual mass yields and CC contents of Lig-CCs and oLig-CCs.

	Theoretical		Experimental		
	Mass yield, %	CC content, mmol/g	Mass yield, %	CC content, mmol/g	**Experimental CC / Theoretical CC**
**SW-KL**	139	2.8	137	2.8	1.0
**HW-OL**	128	2.2	149	3.1	1.4
**HTL-SW-KL**	181	4.5	130	2.7	0.6
**HTL-HW-OL**	176	4.3	130	3.1	0.7
**H_2-SW-KL**	143	3.0	82	3.5	1.2
**H_2-HW-OL**	138	2.8	154	3.3	1.2

* Expected mass yields were calculated following Equation S1, with *M(grafted chain)* corresponding to 100 g/mol:

Equation S1 $$Expected\ mass\ yield = 1 + PhOH\ content\ \times M(grafted\ chain)$$

Table S6. CC contents of Lig-CCs and comparison to initial PhOH contents in the literature.

Reference	Functionalization methodology	Initial PhOH, mmol/g	CC, mmol/g	Actual CC / Expected CC
[2]	ECH+CO_2	3.55	2.01	0.8
		3.90	1.78	0.6
[5]	GC+DMC	1.74	1.5	1.0
[6]	GC+DMC	3.53	2.05	0.8
[7]	GC+DMC	1.83	0.91	0.6

SEC, TGA, and DSC characterization of Lig-CCs and oLig-CCs

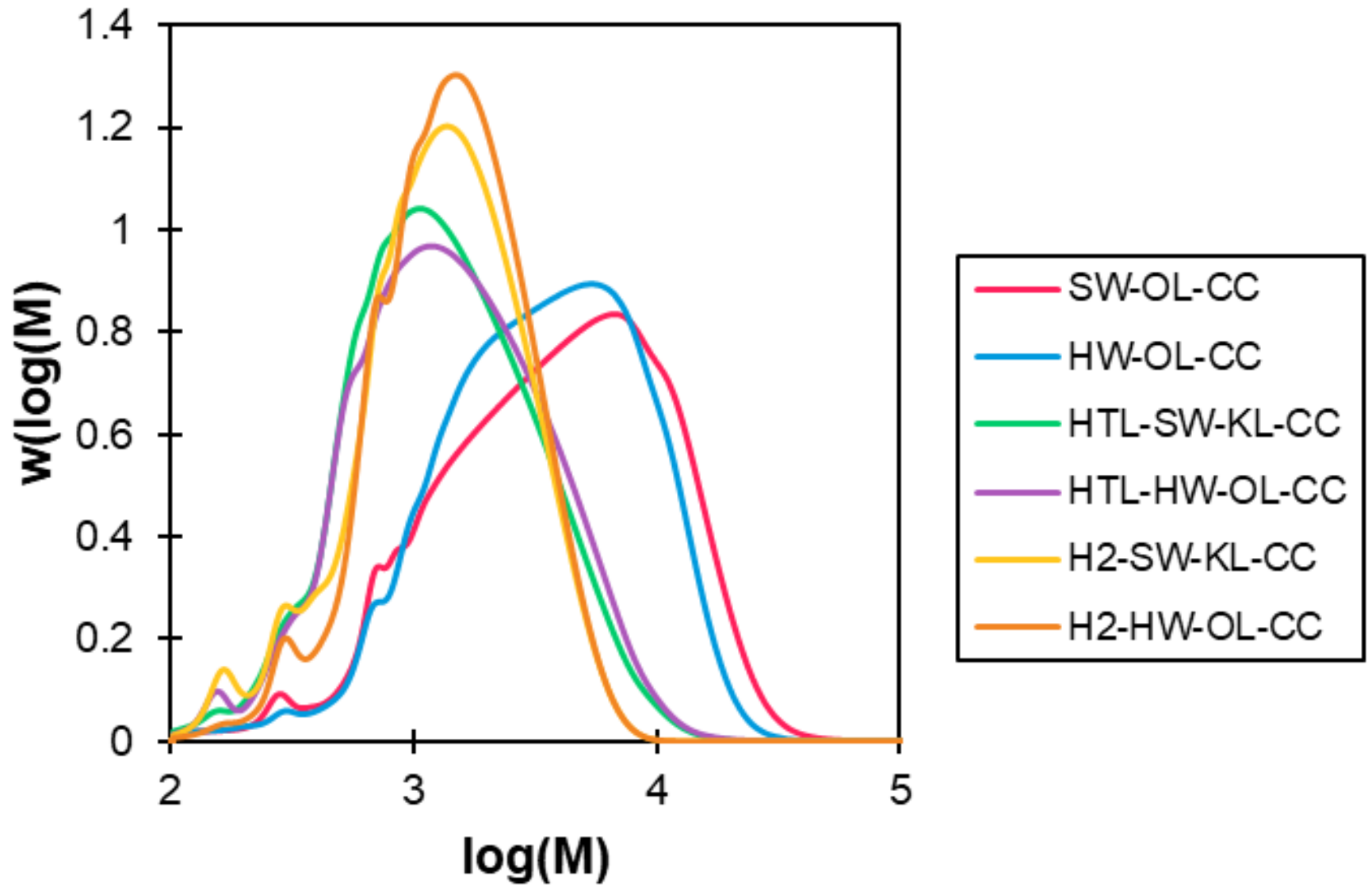

Figure S15. SEC chromatograms for acetylated Lig-CCs and oLig-CCs normalized on total signal. Detector: UV transmission. Solvent: THF. Standards PS.

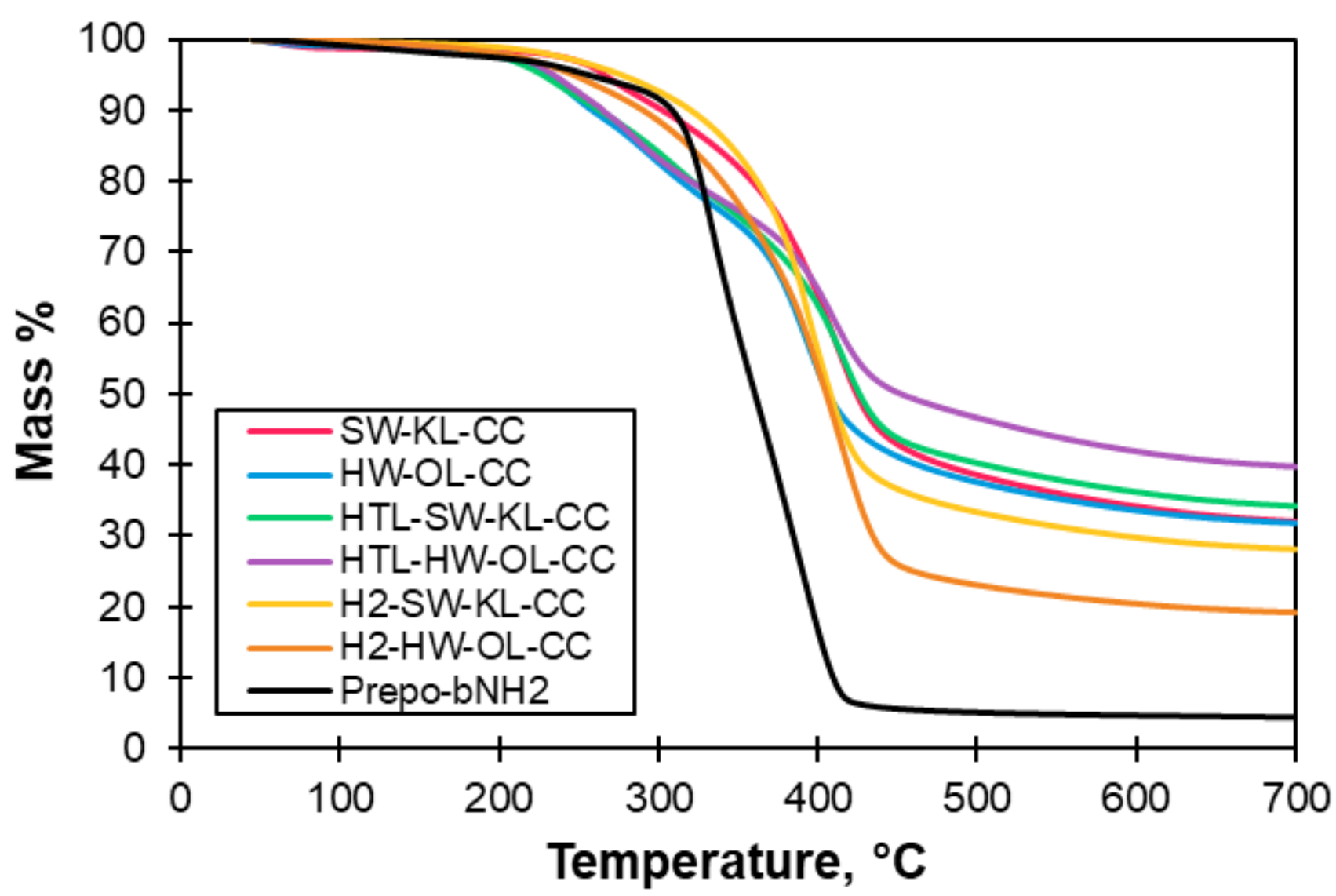

Figure S16. TGA curve for Lig-CCs, oLig-CCs, and Prepo-bNH$_2$. Temperature ramp: 20 °C/min. Gas: N_2.

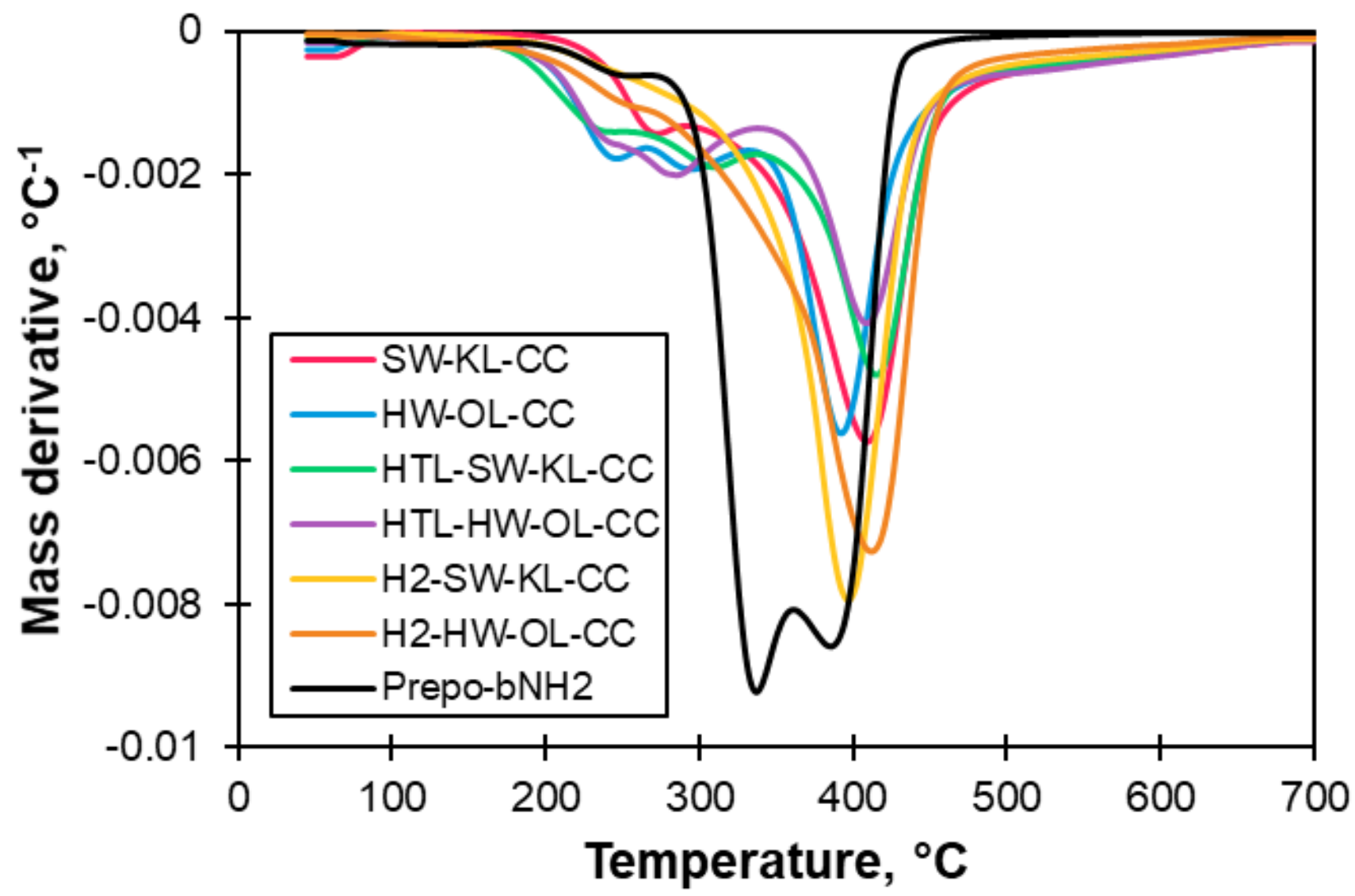

Figure S17. DTG curve for Lig-CCs, oLig-CCs, and Prepo-bNH$_2$. Temperature ramp: 20 °C/min. Gas: N_2.

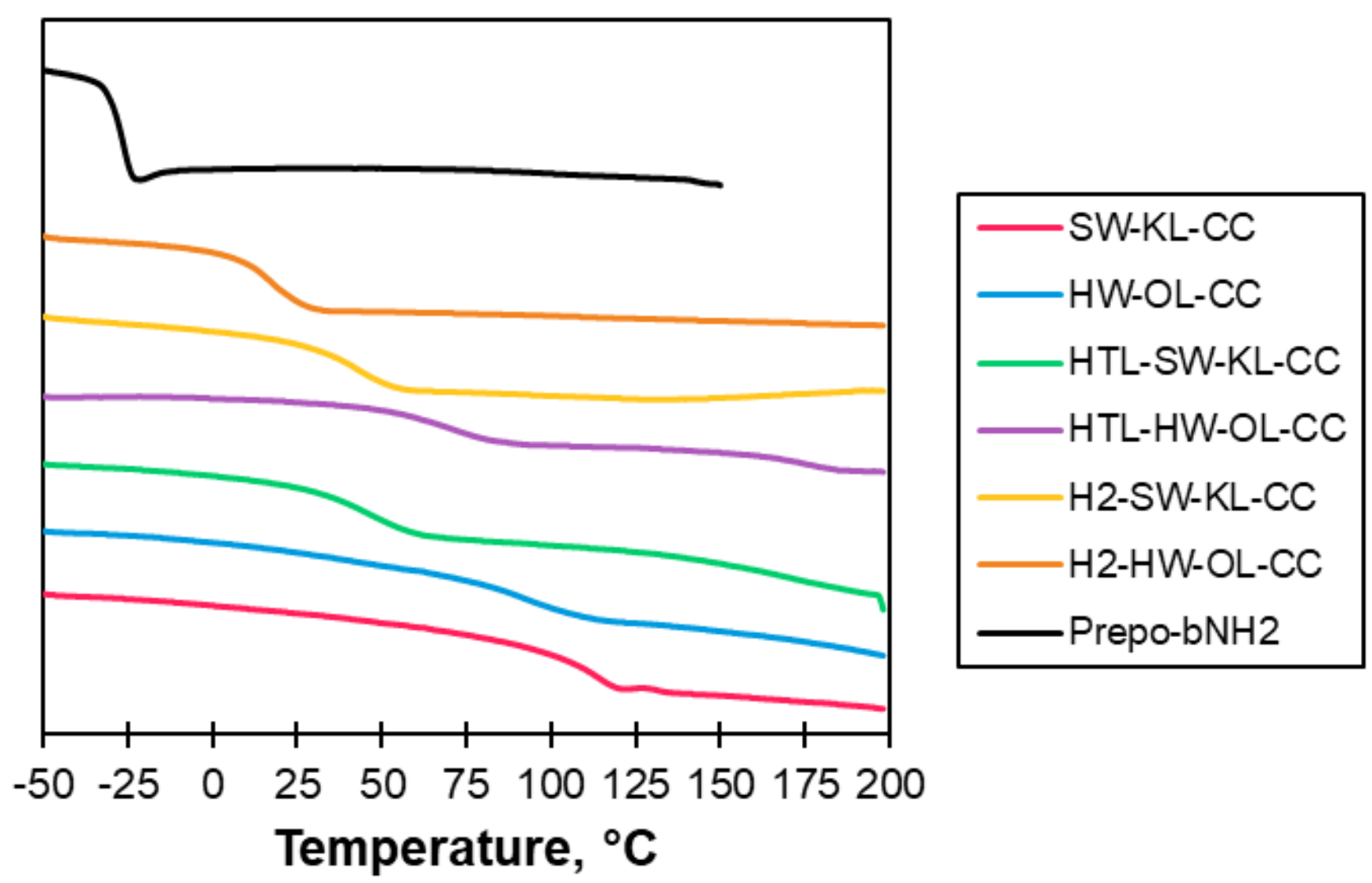

Figure S18. DSC curve for Lig-CCs, oLig-CCs, and Prepo-bNH$_2$. Temperature ramp: 10 °C/min.

Table S7. SEC, TGA, and DSC data for Lig-CCs and oLig-CCs.

	M_n, g/mol	M_w, g/mol	*Đ*	$T_{5\%}$, °C	T_d, °C	%wt residue at 700°C	T_g, °C
**SW-KL-CC**	2020	5940	2.94	266	409	273	111
**HW-OL-CC**	2008	4900	2.44	230	392	245-295	94
**HTL-SW-KL-CC**	921	1903	2.07	227	415	245-310	41
**HTL-HW-OL-CC**	947	2062	2.18	234	409	245-285	63
**H_2-SW-KL-CC**	912	1675	1.84	277	397	-	41
**H_2-HW-OL-CC**	1083	1776	1.64	247	412	-	17

PHUs synthesis

^{1}H NMR of PEG derivatives

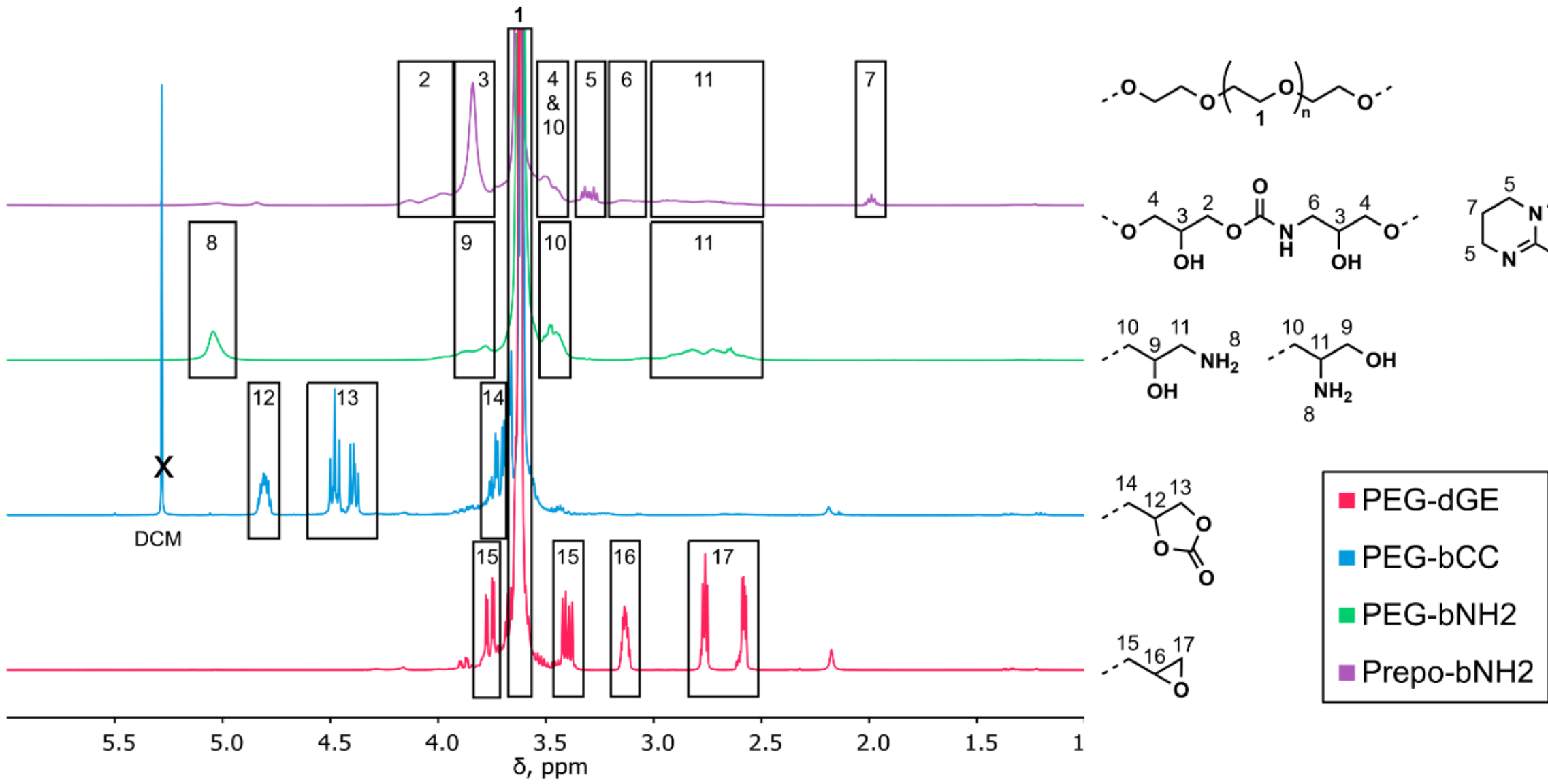

Figure S19. ^{1}H NMR of PEG-dGE, PEG-bCC, PEG-bNH$_2$, and Prepo-bNH$_2$. Solvent: $CDCl_3$.

Quantitative NMR for the evaluation of CC and NH$_2$ groups in PEG derivatives

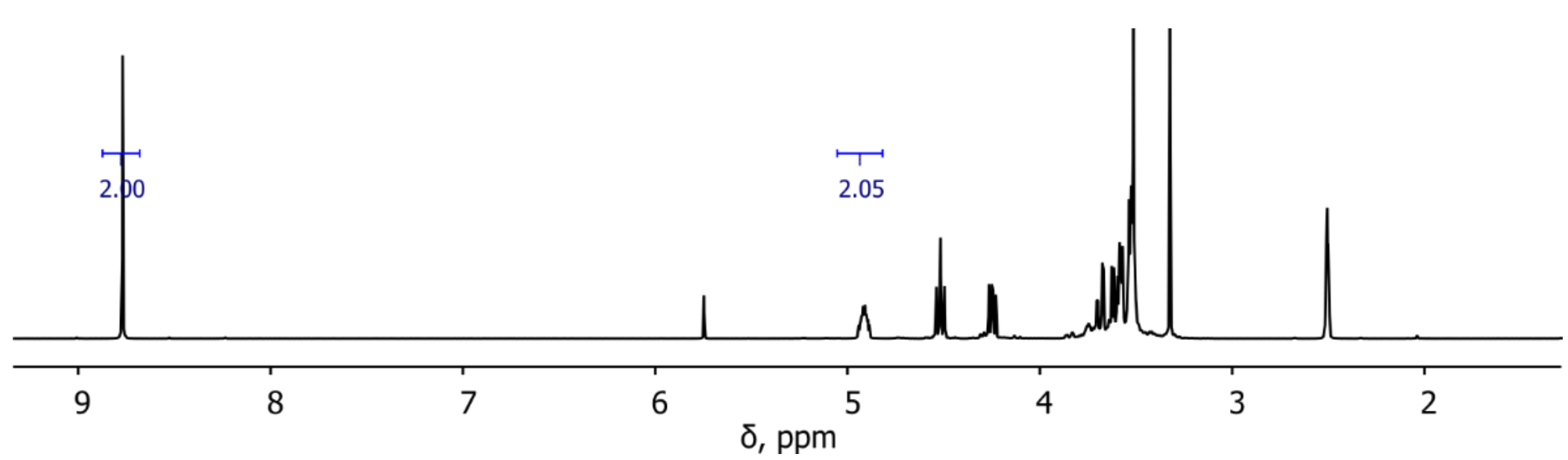

Figure S20. Quantitative ^{1}H NMR of PEG-bCC (32.2 mg). Solvent: DMSO-d_6. Internal standard: 3,4,5-trichloropyridine (TCP), δ (2H, 8.77 ppm). Integration of the signal of proton n°12 (see 1H NMR, **Figure S19**) leads to a content in cyclocarbonates of 3.2 ppm, which corresponds to a full conversion of epoxy groups to CC.

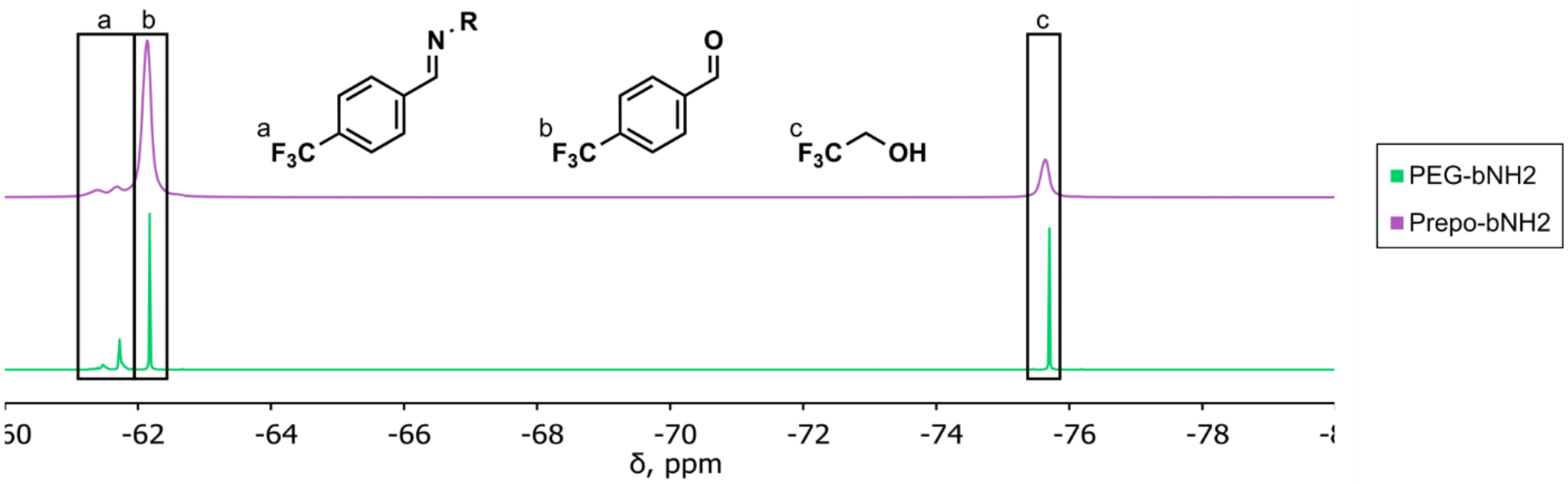

Figure S21. Quantitative ^{19}F NMR of PEG-bNH$_2$ and Prepo-bNH$_2$. Solvent: DMSO-d_6. para-(trifluoromethyl)benzaldehyde (reactant) δ -62.2 ppm, 2,2,2-trifluoroethanol (internal standard) δ -75.7 ppm, imines (from primary amines) δ -61.8 ppm. Integration of imine signal for PEG-bNH$_2$ leads to a primary amine content of 3.2 mmol/g, which corresponds to a full conversion of epoxy groups to hydroxyamines. For Prepo-bNH$_2$, the broadening of the signals did not allow the quantification of amines despite their visible signal between -61.5 and -65 ppm.

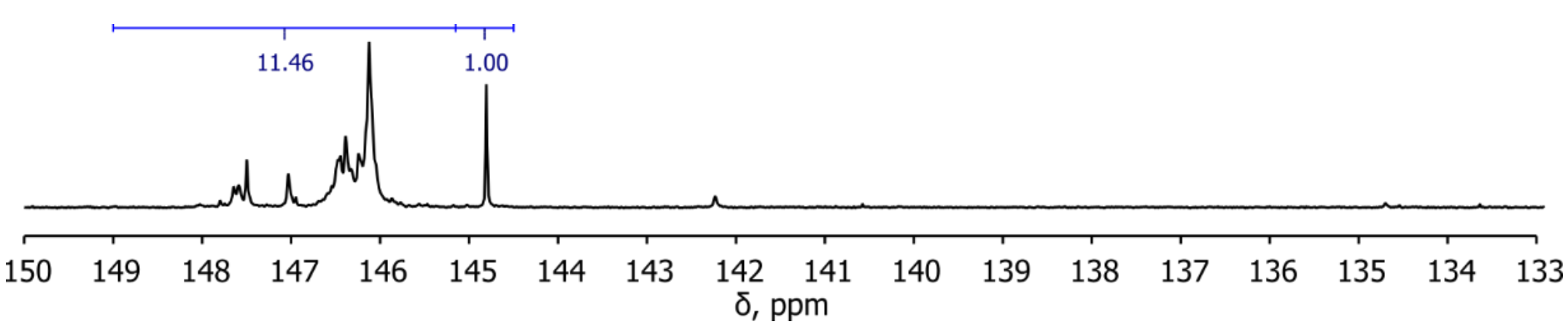

Figure S22. Quantitative ^{31}P NMR of Prepo-bNH$_2$ (35.4 mg). Solvent: $CDCl_3$/Pyridine 1/1.6.

The integration of the aliphatic alcohol signal for PEG-bNH$_2$ resulted in an AlOH content of 3.2 mmol/g, corresponding to a full reaction of PEG-bCC with PEG-bNH$_2$. Thus, Prepo-bNH$_2$ would have an amine content of 1.37 mmol/g.

PHU reaction mixture mixing

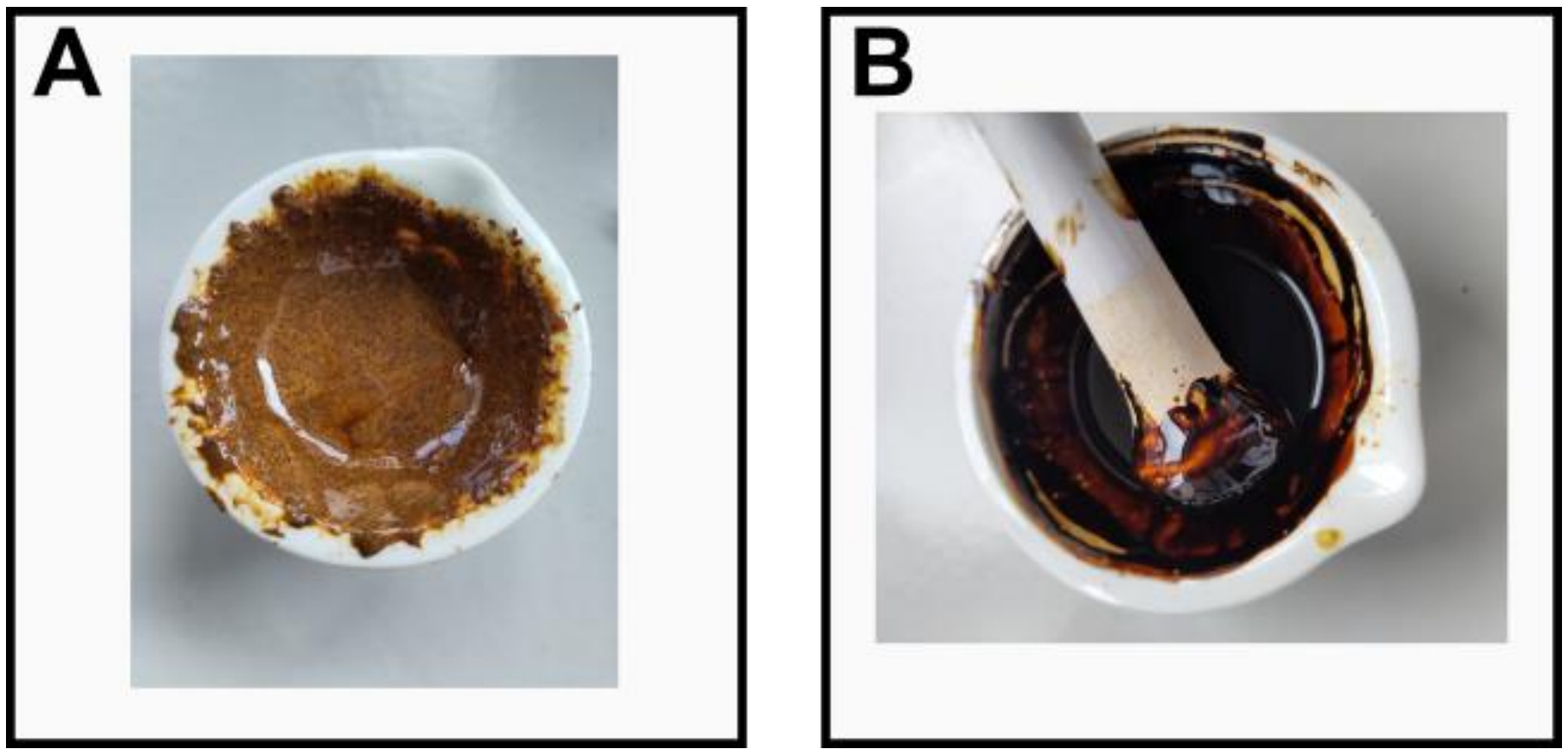

Figure S23. (A) Reaction mixture for PHU-SW-KL (Prepo-bNH_2 & SW-KL-CC) after mixing at room temperature. (B) Same reaction mixture after mixing at room temperature with acetone.

FTIR along PHUs synthesis

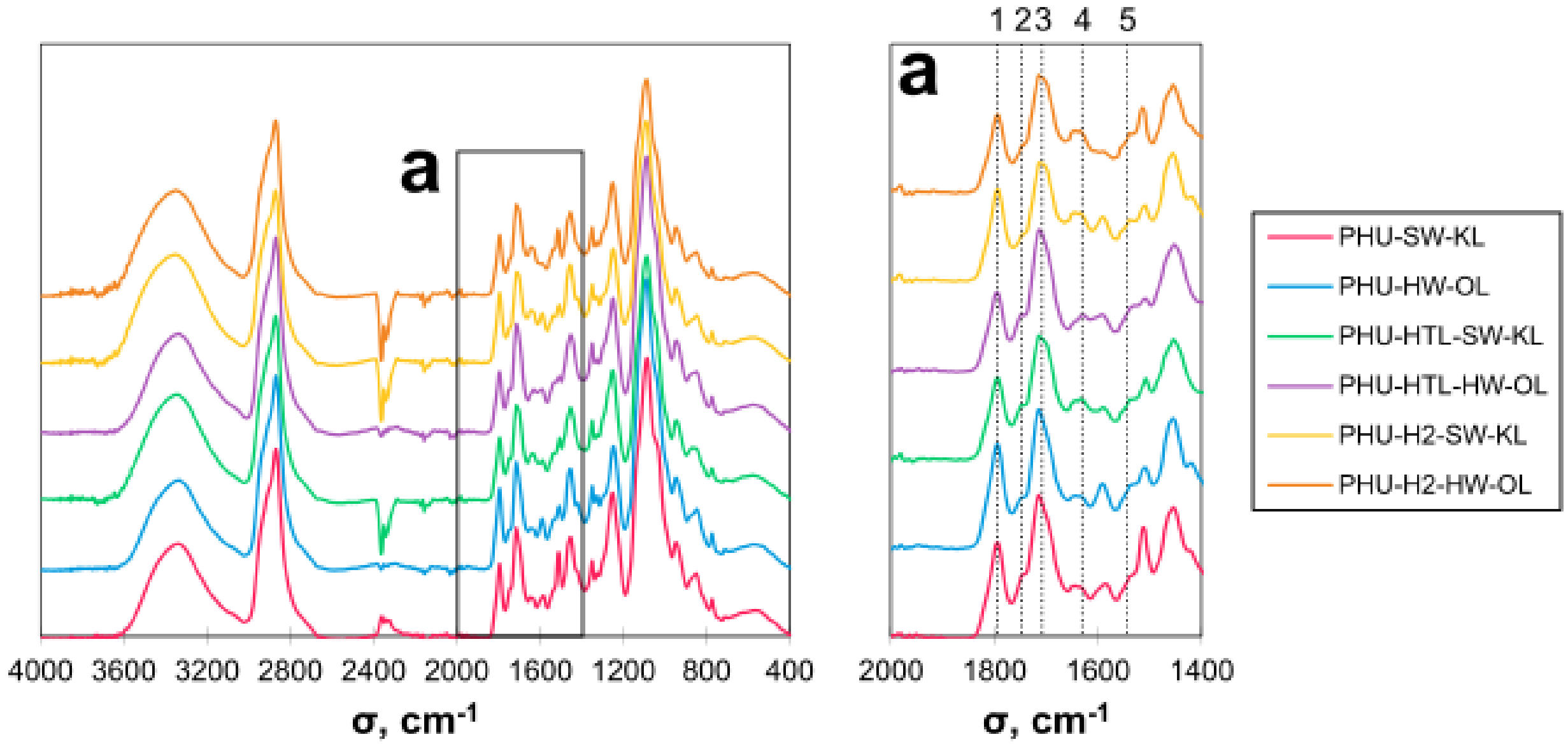

Figure S24. FTIR of the reaction mixture of all PHUs after curing at 80 °C for 4 h, and 120 °C for 4h. (a) Zoom of the region 1400-2000 cm^{-1}. Bands attribution: (1) $C=O_{CC}$ band, 1790 cm^{-1}. (2) Secondary $C=O_{CC}$ band, 1740 cm^{-1}. (3) $C=O_{urethane}$ band, 1710 cm^{-1}. (4) $N-H_{amine}$ band, 1630 cm^{-1}, (5) $N-H_{urethane}$ band, 1520 cm^{-1}.

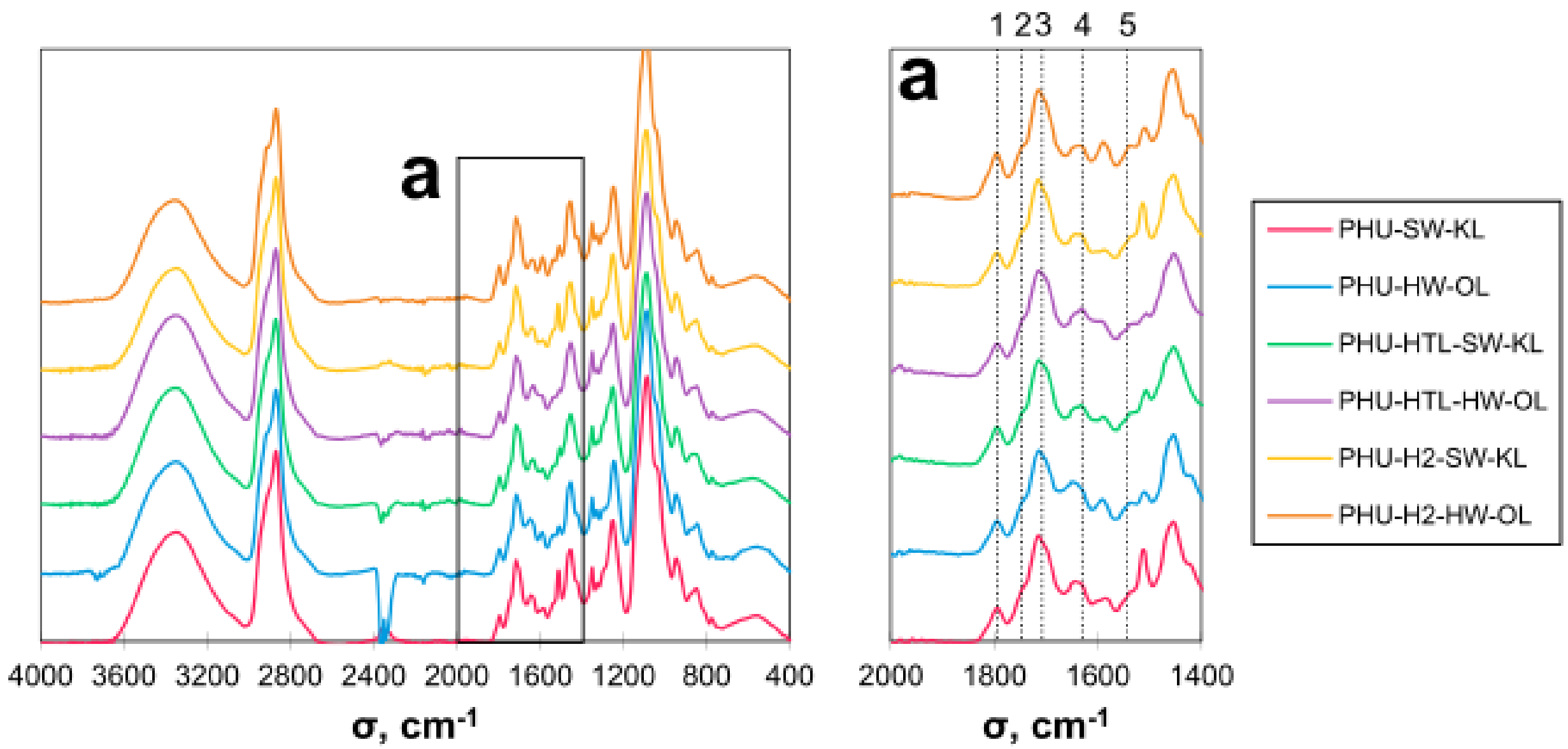

Figure S25. FTIR of the reaction mixture of all PHUs after curing at 80 °C for 4 h, 120 °C for 4h, and 160 °C for 2 h (in the hot press for Lig-based PHUs). (a) Zoom of the region 1400-2000 cm^{-1}. Bands attribution: (1) $C{=}O_{CC}$ band, 1790 cm^{-1}. (2) Secondary $C{=}O_{CC}$ band, 1740 cm^{-1}. (3) $C{=}O_{urethane}$ band, 1710 cm^{-1}. (4) $N\text{-}H_{amine}$ band, 1630 cm^{-1}, (5) $N\text{-}H_{urethane}$ band, 1520 cm^{-1}.

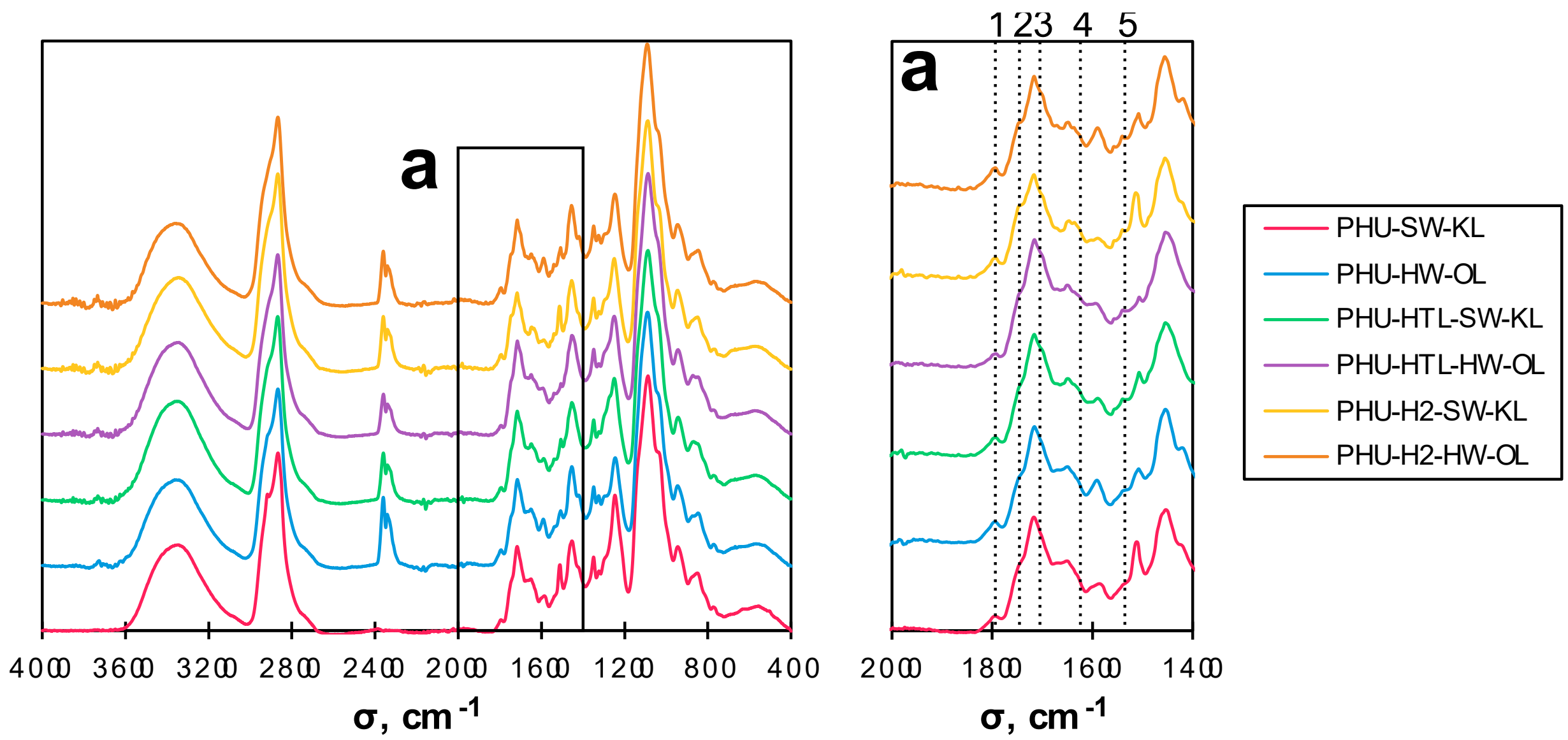

Figure S26. FTIR of the reaction mixture of all PHUs after curing at 80 °C for 4 h, 120 °C for 4h, and 160 °C for 6 h. (a) Zoom of the region 1400-2000 cm^{-1}. Bands attribution: (1) $C=O_{CC}$ band, 1790 cm^{-1}. (2) Secondary $C=O_{CC}$ band, 1740 cm^{-1}. (3) $C=O_{urethane}$ band, 1710 cm^{-1}. (4) $N\text{-}H_{amine}$ band, 1630 cm^{-1}, (5) $N\text{-}H_{urethane}$ band, 1520 cm^{-1}.

PHUs characterization

Thermal and mechanical properties by TGA, DSC, and DMA

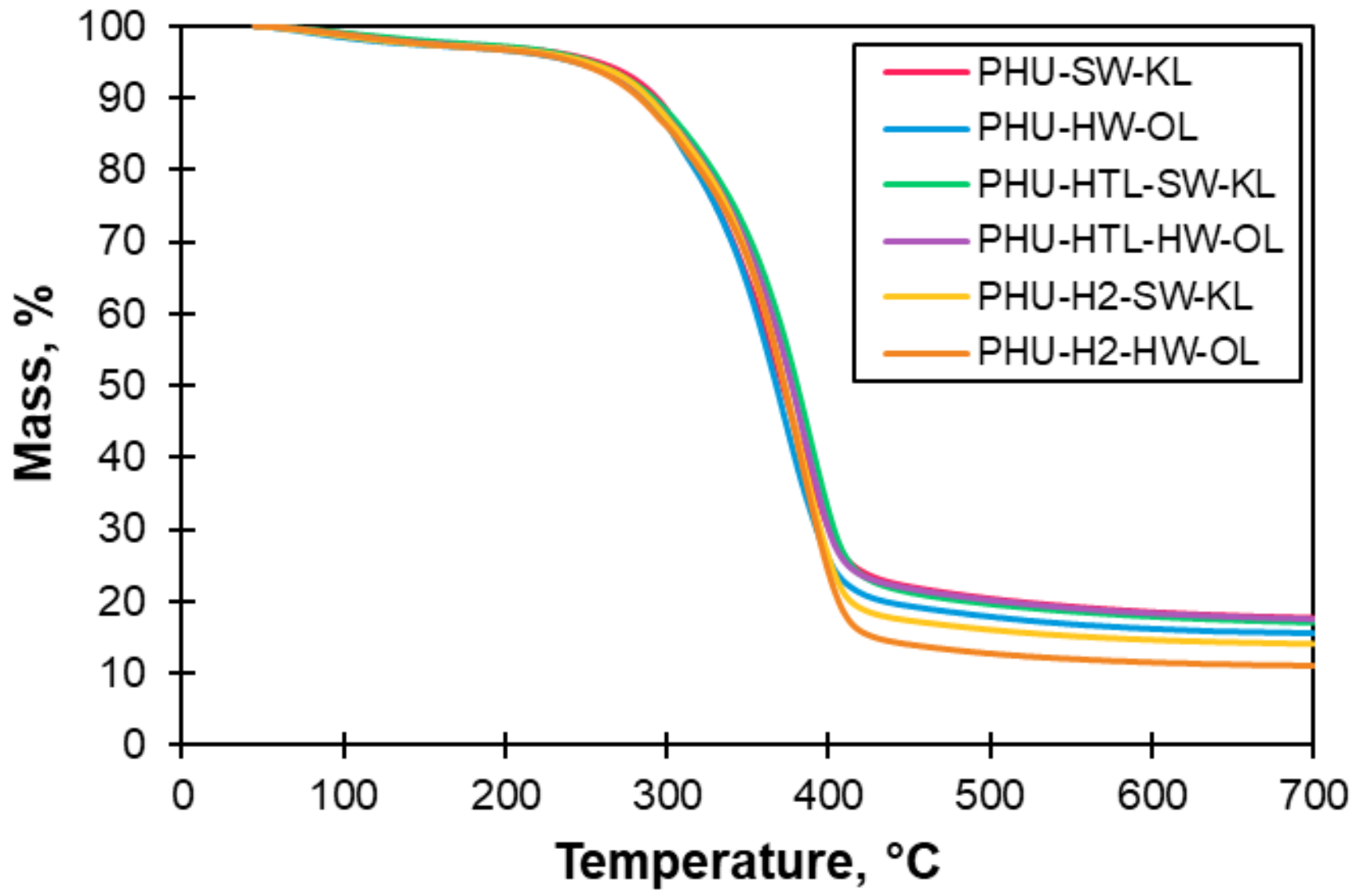

Figure S27. TGA curve for the PHUs. Temperature ramp: 20 °C/min. Gas: N_2.

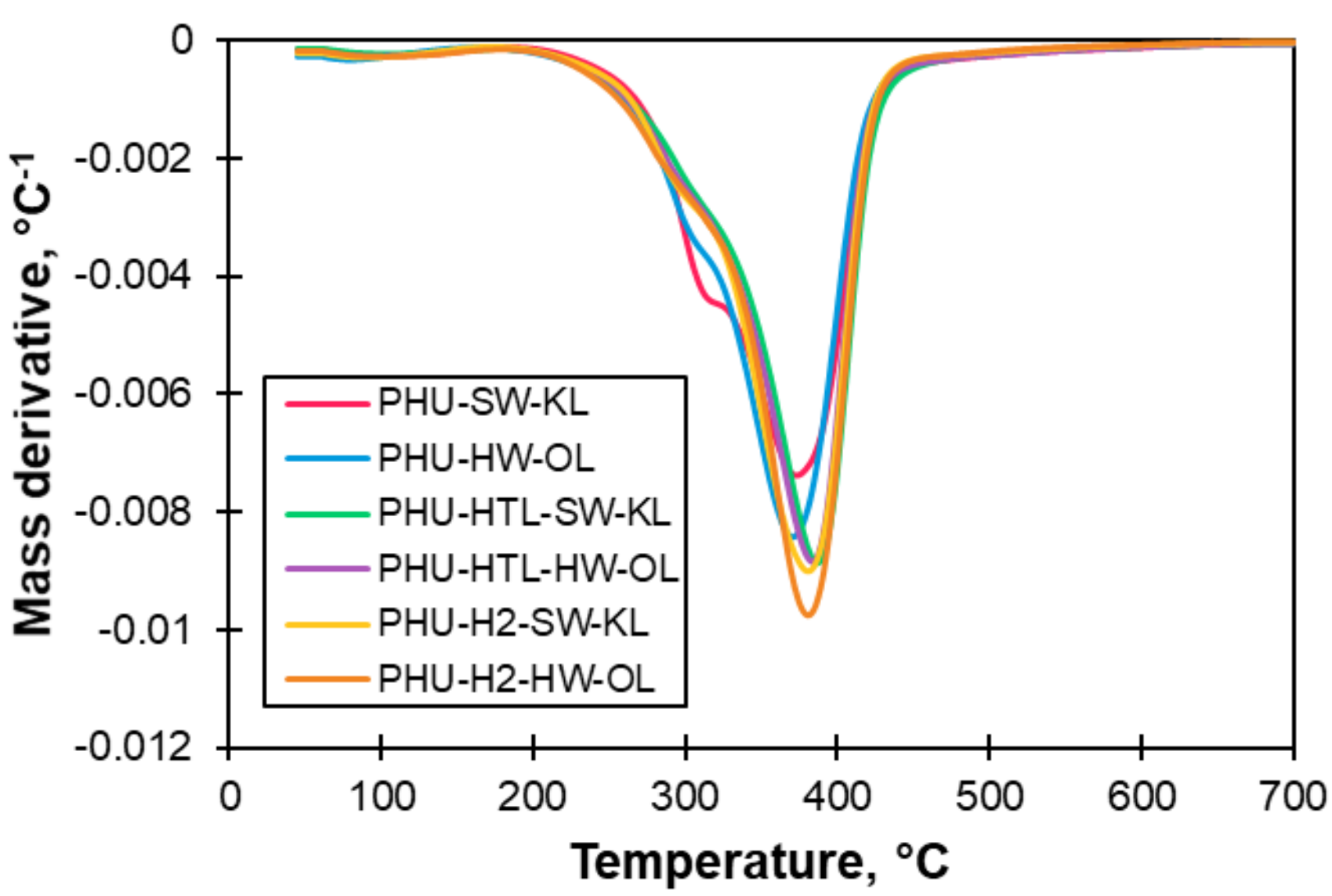

Figure S28. DTG curve for the PHUs. Temperature ramp: 20 °C/min. Gas: N_2.

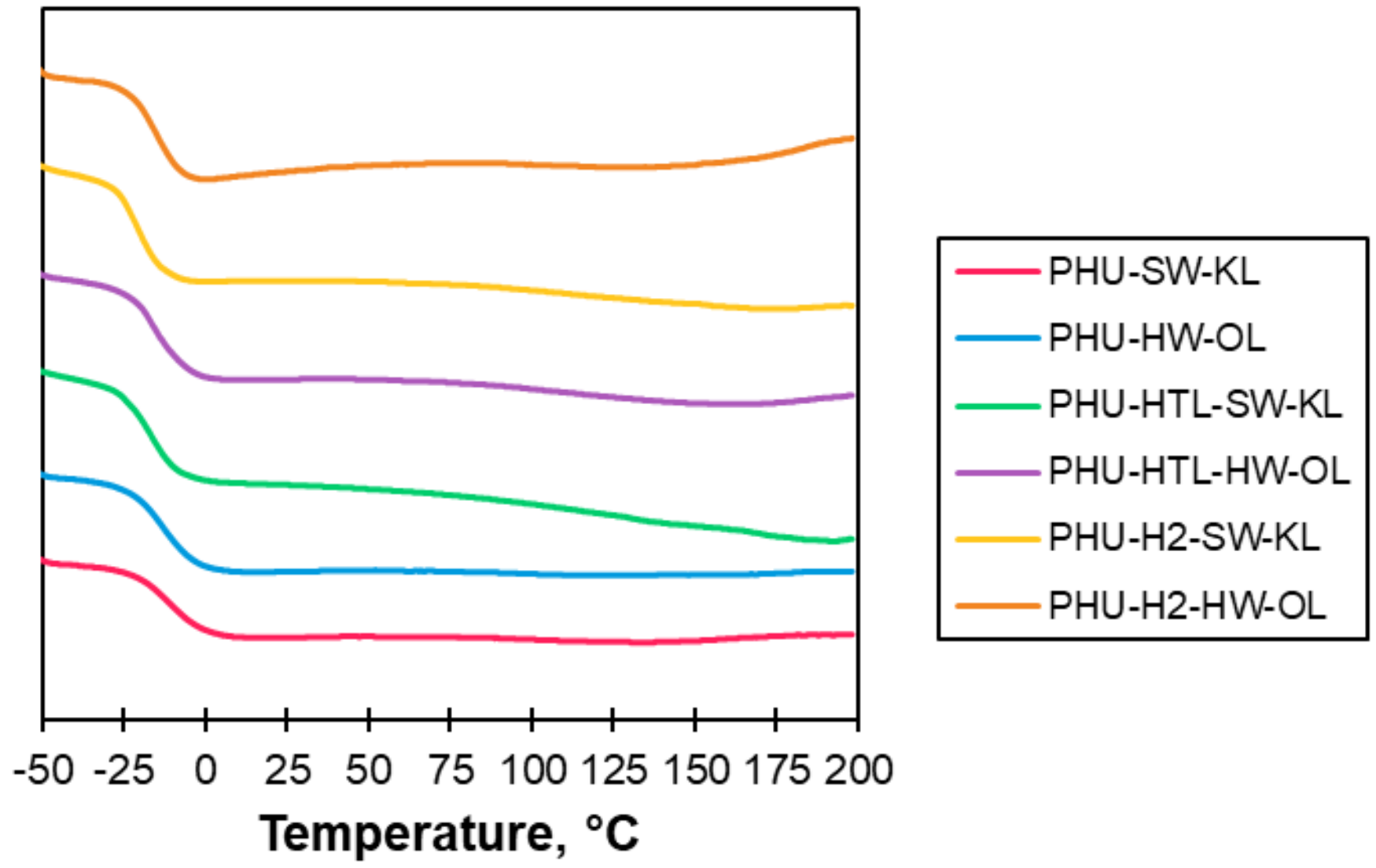

Figure S29. DSC curve for the PHUs. Temperature ramp: 10 °C/min.

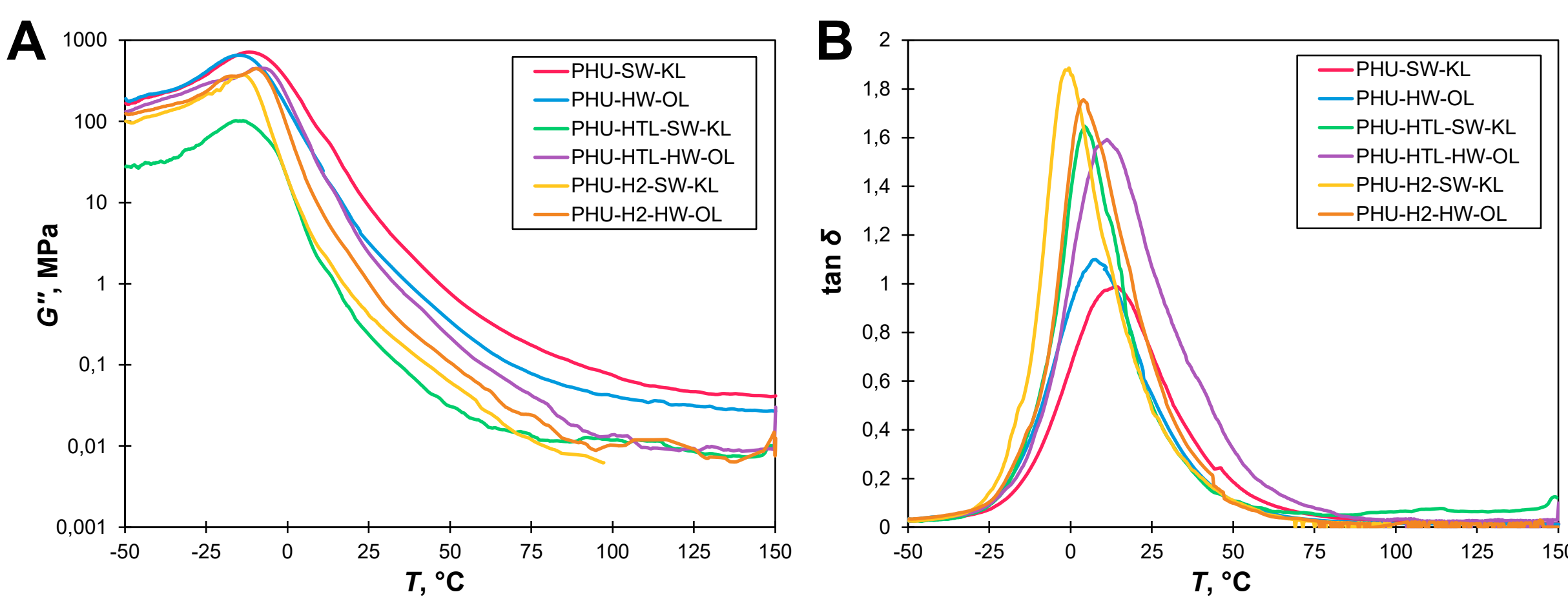

Figure S30. (A) Loss modulus (*G''*) and (B) tan *δ* curves as a function of temperature measured by DMA.

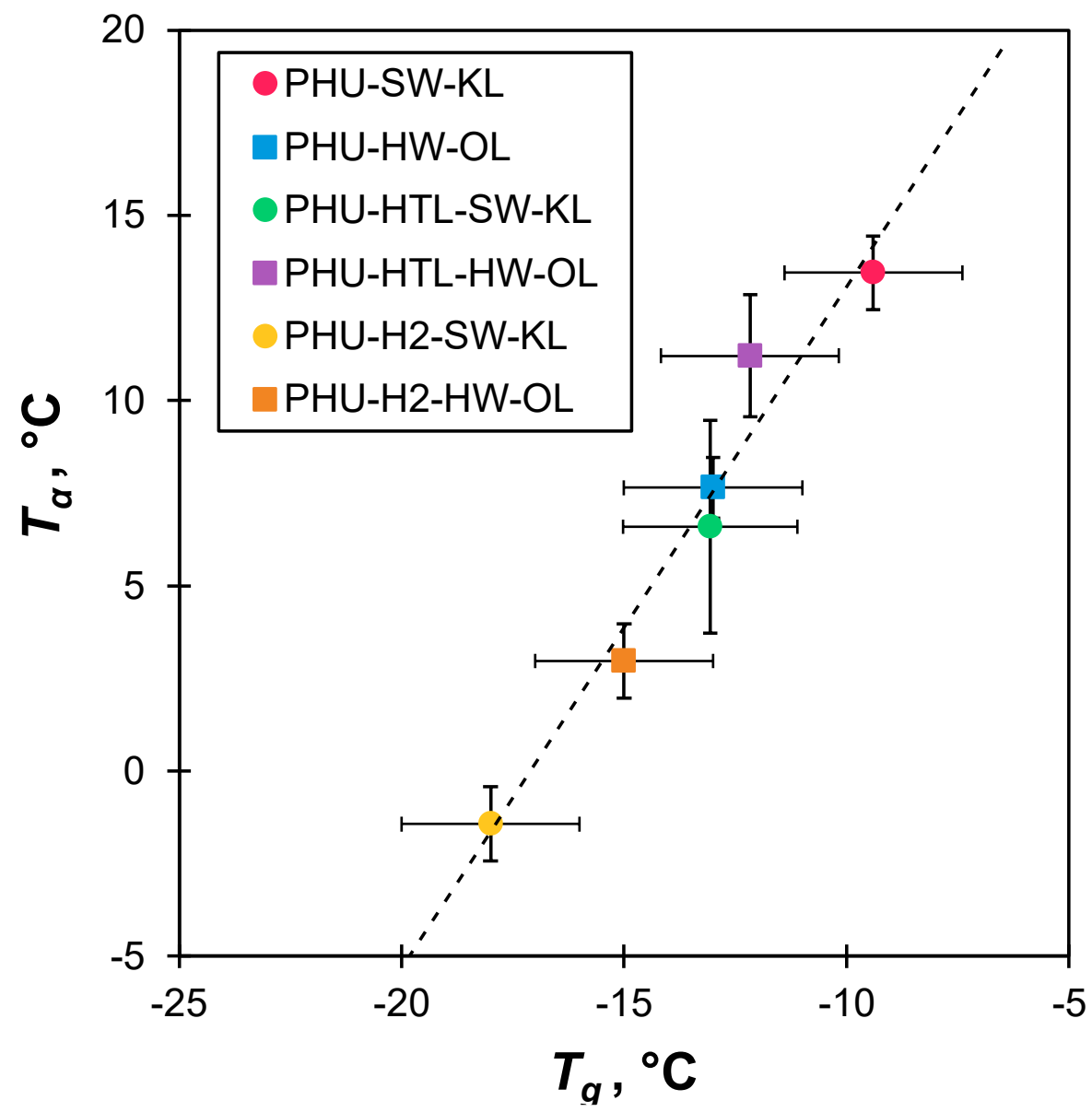

Figure S31. Correlation between T_α measured by DMA and T_g measured by DSC for the PHUs.

Swelling properties

Table S8. PHUs swelling ratios (*SR*) and gel fractions (*GF*) in acetone and water.

	**Swelling ratio in H_2O**	**Swelling ratio in Acetone**	**Gel fraction in H_2O**	**Gel fraction in Acetone**
**PHU-SW-KL**	254 ± 9 ^{A}	29 ± 1 ^{A}	77 ± 2 ^{A}	92 ± 1 ^{A}
**PHU-HW-OL**	288 ± 10 ^{A}	33 ± 1 ^{A}	74 ± 1 ^{A}	86 ± 1 ^{B}
**PHU-HTL-SW-KL**	1020 ± 91 ^{B}	98 ± 5 ^{B}	50 ± 4 ^{B}	74 ± 1 ^{C,D}
**PHU-HTL-HW-OL**	990 ± 86 ^{B}	71 ± 3 ^{C}	56 ± 1 ^{B}	79 ± 1 ^{E}
**PHU-H_2-SW-KL**	1117 ± 89 ^{B}	61 ± 1 ^{D}	57 ± 3 ^{B}	71 ± 1 ^{C}
**PHU-H_2-HW-OL**	732 ± 115 ^{C}	49 ± 3 ^{E}	65 ± 3 ^{C}	76 ± 2 ^{D,E}

Values labelled with different capital letters are significatively different (Tukey's HSD test, $\alpha = 0.05$).

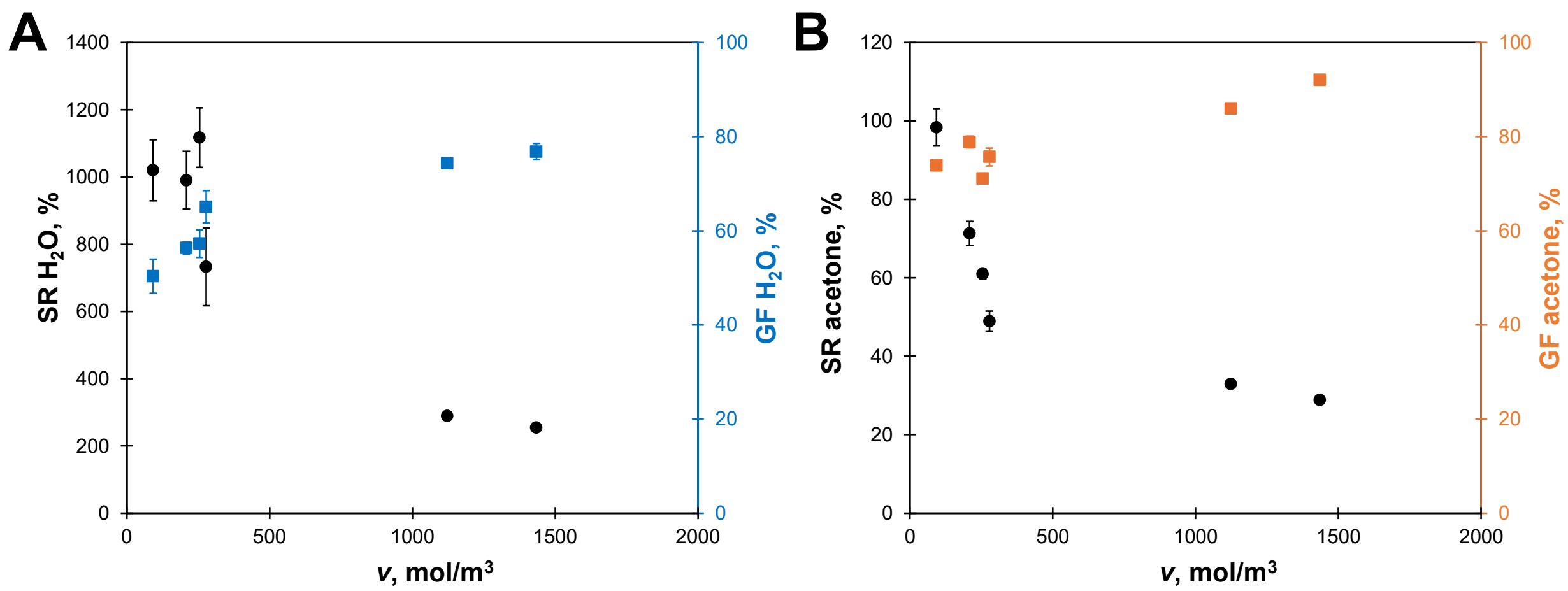

Figure S32. Correlation between the swelling ratio (SR) and gel fraction (GF) of the PHUs and the crosslink density measured by DMA. (A) SR and GF measured in water, (B) SR and GF measured in acetone.

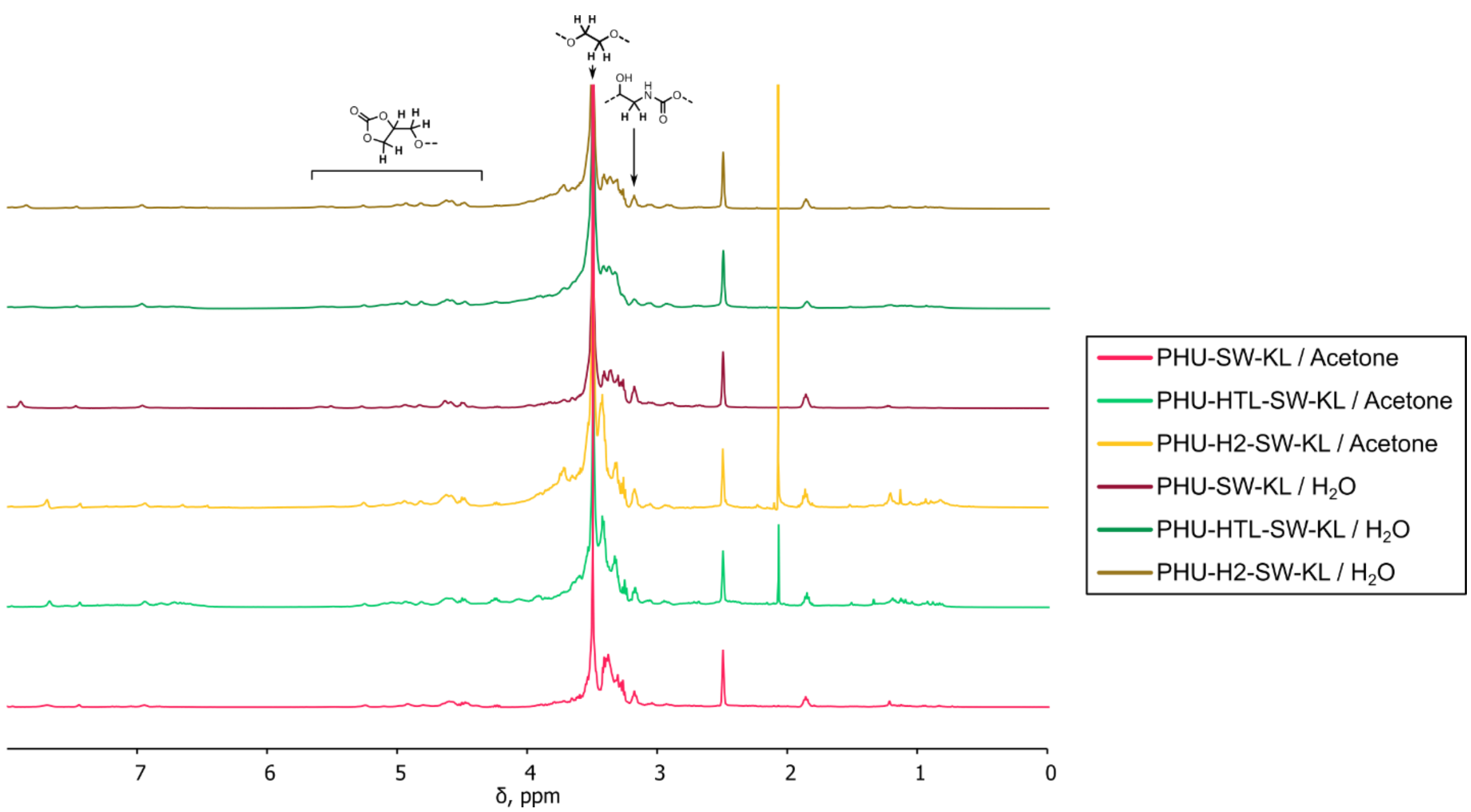

Figure S33. ^{1}H NMR of the soluble fractions of PHUs derived from SW-KL in acetone and water.

Tensile properties

Table S9. Mechanical properties of the PHUs from HW-OL obtained by tensile tests. *E*, *σ* and *ε*, respectively Young's modulus, maximum stress, and maximum strain as given by traction experiments.

	***E*, kPa**	***σ*, kPa**	***ε*, %**
**PHU-SW-KL**	1414 ± 290 A	484 ± 37 A	46 ± 9 A
**PHU-HW-OL**	864 ± 53 B	461 ± 45 A	63 ± 2 A
**PHU-HTL-SW-KL**	50 ± 11 C	92 ± 3 B	217 ± 22 B
**PHU-HTL-HW-OL**	79 ± 25 C	79 ± 11 B	166 ± 28 B,C
**PHU-H_2-SW-KL**	60 ± 22 C	88 ± 8 B	182 ± 43 B,C
**PHU-H_2-HW-OL**	81 ± 6 C	92 ± 8 B	135 ± 10 C

Values labelled with different capital letters are significatively different (Tukey's HSD test, α = 0.05).

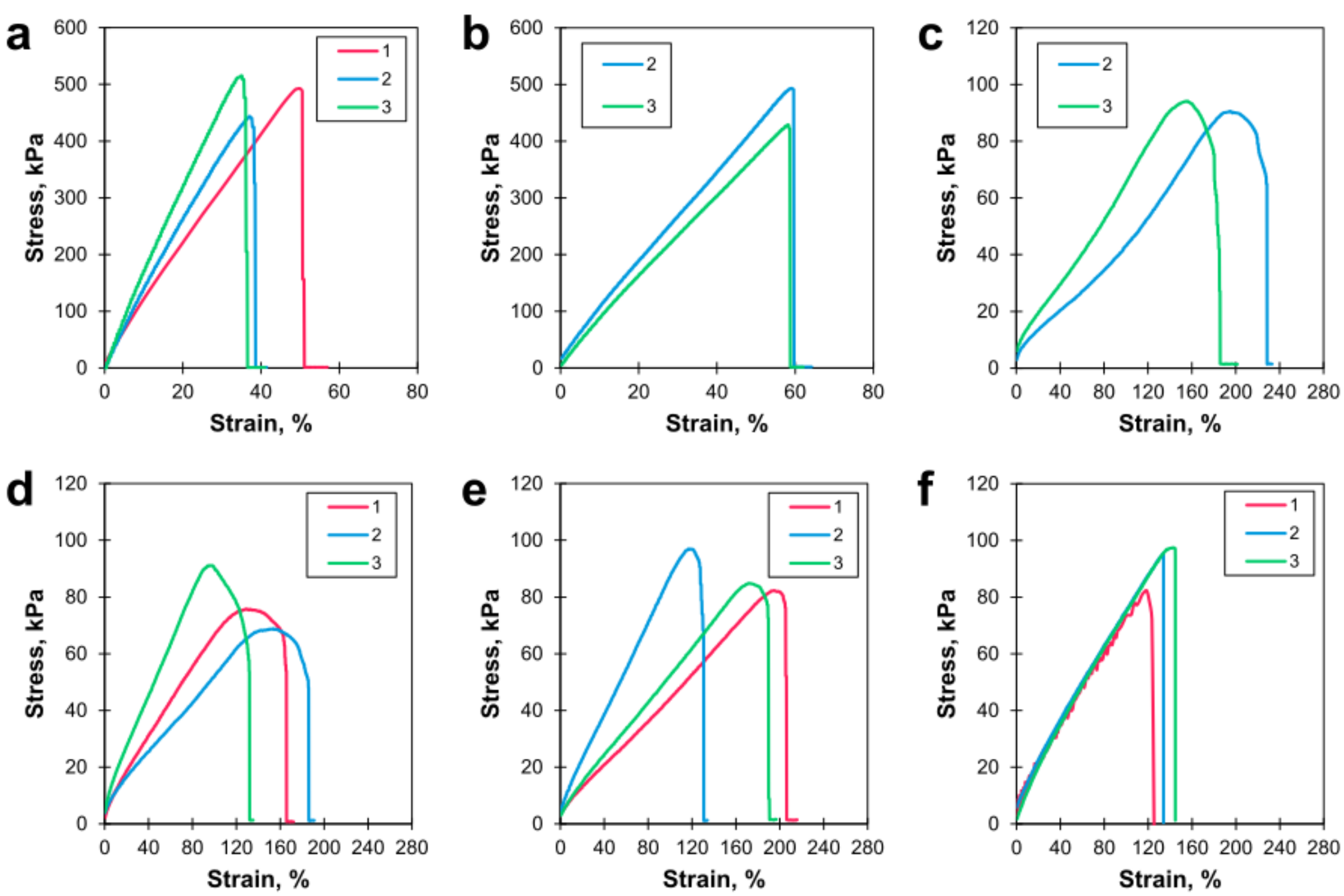

Figure S34. Uniaxial Tensile Stress-Strain curves for (a) PHU-SW-KL, (b) PHU-HW-OL, (c) PHU-HTL-SW-KL, (d) PHU-HTL-HW-OL, (e) PHU-H_2-SW-KL, and (f) PHU-H_2-HW-OL.

Mechanical properties comparison with literature

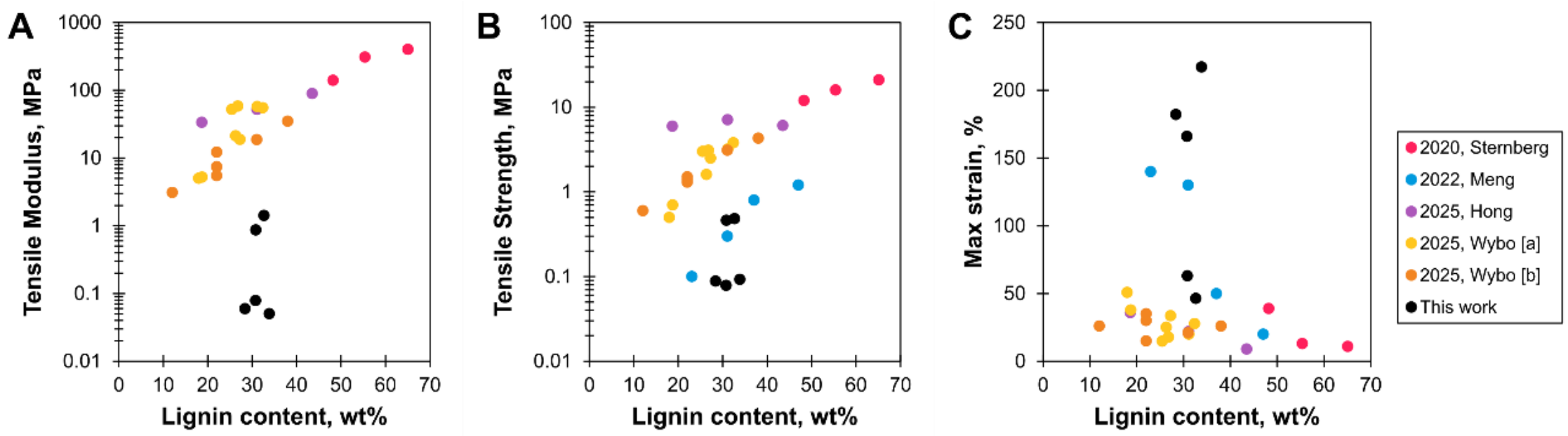

Figure S35. Reported lignin-based NIPUs tensile properties in the literature. (A) Tensile modulus, (B) Tensile Strength, and (C) Max strain. For each study, the different dots represent the different materials. References: 2020, Sternberg;[6] 2022, Meng;[8] 2025, Hong;[9] 2025, Wybo [a];[10] 2025, Wybo [b][11]

Supplementary information references

(1) Smit, A. T.; Dezaire, T.; Riddell, L. A.; Bruijnincx, P. C. A. Reductive Partial Depolymerization of Acetone Organosolv Lignin to Tailor Lignin Molar Mass, Dispersity, and Reactivity for Polymer Applications. *ACS Sustainable Chem. Eng.* **2023**, *11* (15), 6070–6080. https://doi.org/10.1021/acssuschemeng.3c00617.

(2) Salanti, A.; Zoia, L.; Orlandi, M. Chemical Modifications of Lignin for the Preparation of Macromers Containing Cyclic Carbonates. *Green Chem.* **2016**, *18* (14), 4063–4072. https://doi.org/10.1039/C6GC01028H.

(3) Yang, Y.; Cao, H.; Liu, R.; Wang, Y.; Zhu, M.; Su, C.; Lv, X.; Zhao, J.; Qin, P.; Cai, D. Fabrication of Ultraviolet Resistant and Anti-Bacterial Non-Isocyanate Polyurethanes Using the Oligomers from the Reductive Catalytic Fractionated Lignin Oil. *Industrial Crops and Products* **2023**, *193*, 116213. https://doi.org/10.1016/j.indcrop.2022.116213.

(4) Yamini, G.; Shakeri, A.; Zohuriaan-Mehr, M. J.; Kabiri, K. Cyclocarbonated Lignosulfonate as a Bio-Resourced Reactive Reinforcing Agent for Epoxy Biocomposite: From Natural Waste to Value-Added Bio-Additive. *Journal of CO2 Utilization* **2018**, *24*, 50–58. https://doi.org/10.1016/j.jcou.2017.12.007.

(5) Kühnel, I.; Saake, B.; Lehnen, R. A New Environmentally Friendly Approach to Lignin-Based Cyclic Carbonates. *Macromolecular Chemistry and Physics* **2018**, *219* (7), 1700613. https://doi.org/10.1002/macp.201700613.

(6) Sternberg, J.; Pilla, S. Materials for the Biorefinery: High Bio-Content, Shape Memory Kraft Lignin-Derived Non-Isocyanate Polyurethane Foams Using a Non-Toxic Protocol. *Green Chem.* **2020**, *22* (20), 6922–6935. https://doi.org/10.1039/D0GC01659D.

(7) Destaso, F. C.; Libretti, C.; Coz, C. L.; Grau, E.; Cramail, H.; Meier, M. A. R. Optimized Synthesis of a High Oleic Sunflower Oil Derived Polyamine and Its Lignin-Based NIPUs. *Green Chem.* **2024**, *27*, 1440–1450. https://doi.org/10.1039/D4GC05645K.

(8) Meng, X.; Zhang, S.; Scheidemantle, B.; Wang, Y.; Pu, Y.; Wyman, C. E.; Cai, C. M.; Ragauskas, A. J. Preparation and Characterization of Aminated Co-Solvent Enhanced Lignocellulosic Fractionation Lignin as a Renewable Building Block for the Synthesis of Non-Isocyanate Polyurethanes. *Industrial Crops and Products* **2022**, *178*, 114579. https://doi.org/10.1016/j.indcrop.2022.114579.

(9) Hong, H.; Li, Z.; Wu, H.; Wang, D.; Liu, L.; Luo, W.; Yang, T.; Yang, K.; Yao, J. Polyphenol Lignin Derived Non-Isocyanate Polyurethane with Mechanically Robust and Photothermal Conversion Performances. *International Journal of Biological Macromolecules* **2025**, *310*, 143259. https://doi.org/10.1016/j.ijbiomac.2025.143259.

(10) Wybo, N.; Cherasse, E.; Duval, A.; Avérous, L. Unlocking Sustainable, Aromatic, and Versatile Materials through Transurethanization: Development of Non-Isocyanate Polyurethanes from Lignins. *J. Mater. Chem. A* **2025**, *13* (16), 11557–11572. https://doi.org/10.1039/D4TA08582E.

(11) Wybo, N.; Duval, A.; Avérous, L. Unlocking the Potential of Lignin-Based Polyhydroxyurethanes: Insights into Kinetics, Physical Behavior, and Recyclability. *Materials Today Sustainability* **2025**, *30*, 101117. https://doi.org/10.1016/j.mtsust.2025.101117.
